# Mutant p53 gains RNA-binding activity to reprogram translation and mitochondrial function

**DOI:** 10.1016/j.celrep.2026.117856

**Published:** 2026-08-18

**Authors:** Wuyue Zhou, Alice Long, Cameron J. Douglas, Dalila Gallegos, James M. Burke, David W.C. MacMillan, Ezgi Hacisuleyman, Ciaran P. Seath

**Affiliations:** 1 Department of Chemistry and Chemical Biology, Cornell University, Ithaca, NY 14850, USA; 2 Department of Chemistry, Wertheim UF Scripps, Jupiter, FL 33458, USA; 3 Skaggs Graduate School, The Scripps Research Institute, Jupiter, FL 33458, USA; 4 Department of Chemistry, Princeton University, Princeton, NJ, USA; 5 Department of Molecular Medicine, Wertheim UF Scripps, Jupiter, FL 33458, USA; 6 These authors contributed equally; 7 Lead contact

## Abstract

Tumor suppressor p53 is a transcription factor mutated in ~50% of cancers. Somatic mutations in the DNA-binding domain abolish tumor suppression and thus lead to a loss-of-function activity, while a small subset of recurrent “hotspot” mutants have been shown to confer gain-of-function activities. Using an intein-based **μ**Map photoproximity labeling approach, we map the interactomes of five hotspot mutants and find that mutant p53 (mut p53) acquires interactions with RNA-binding proteins, shifts toward the cytoplasm, and shows increased proximity to structured RNA and RNA-binding proteins. CLIP (Cross-linking and immunoprecipitation) experiments show that mut p53 possesses an RNA-binding motif and is enriched at 3′UTRs, promoting ribosomal localization and localization at the mitochondrial surface. Using ribosome profiling, we further show that mut p53 alters translation and promotes changes in miRNA processing and mitochondrial function, providing a mechanistic rationale for historically reported but poorly understood phenotypes.

## In brief

Zhou et al. show that cancer-associated DNA-binding domain mutations promote the RNA-binding functions of p53 protein. Mutant p53 engages 3′UTRs at ribosomes and mitochondria, increasing translation of mitochondrial mRNAs—a feature gained across G245S and R273H hotspot mutants, supported by ribosome profiling and pan-cancer tumor data.

## Graphical abstract

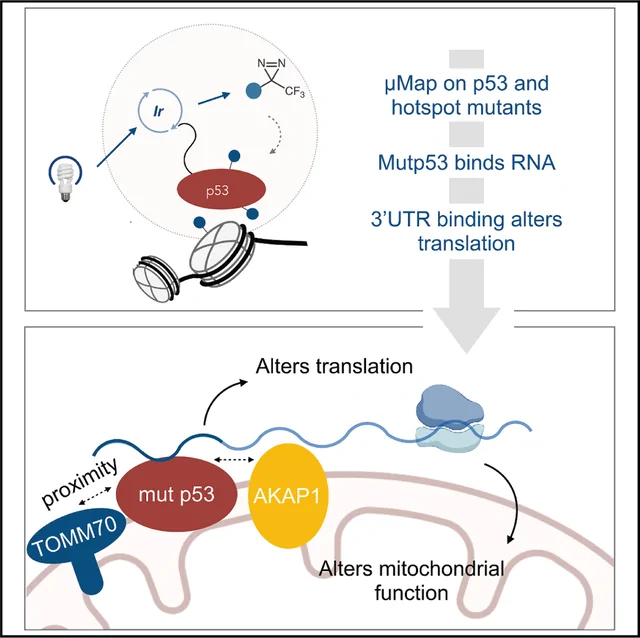

## INTRODUCTION

**Figure 1.**
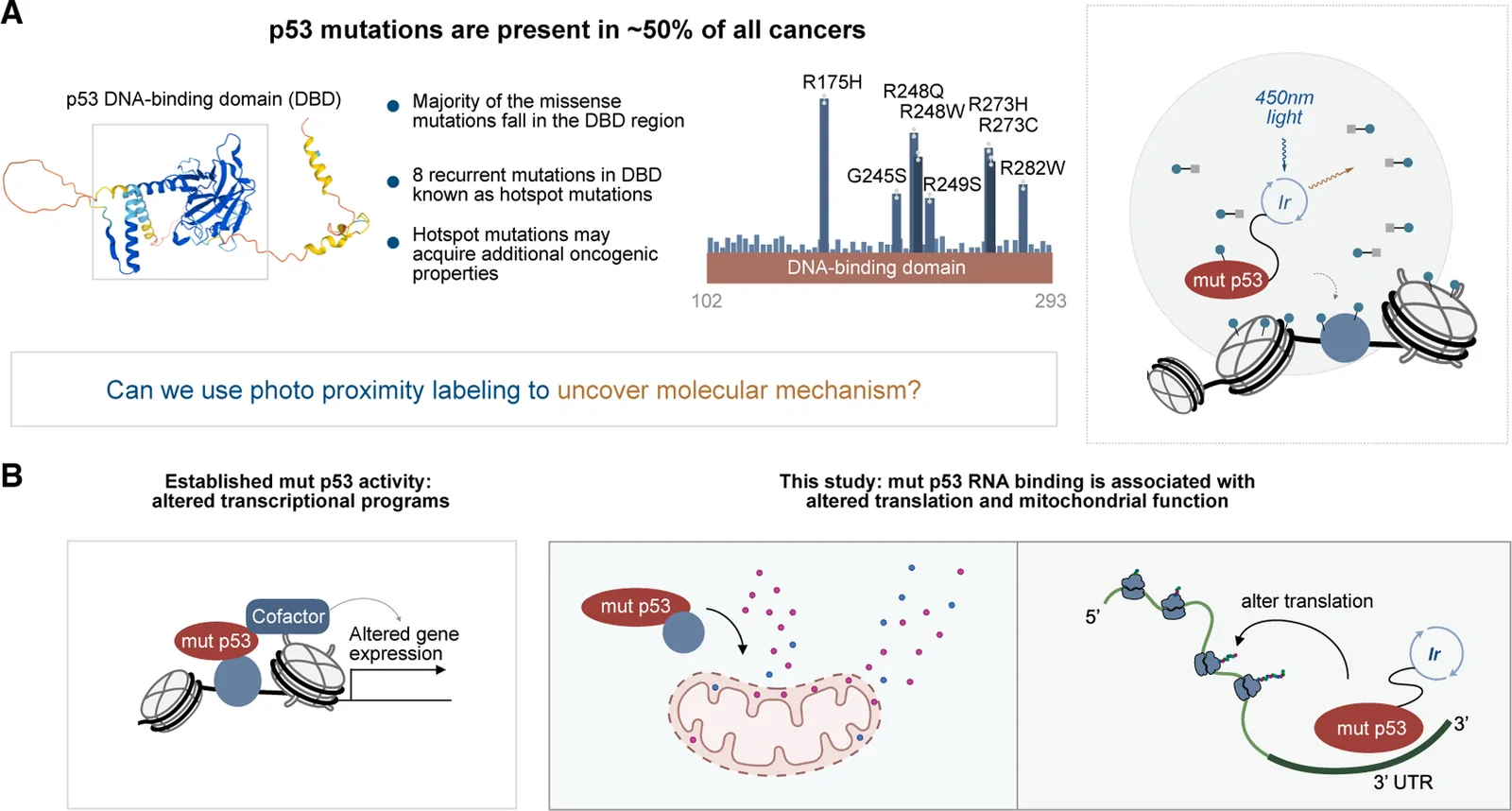
Unraveling mutant p53 function with μMap labeling Mutated p53 has been found in about 50% of all human tumors, and the majority of the missense mutations localizes to the DNA-binding domain (DBD). (A) A recurrent subset of these mutations is termed “hotspot mutations”. In this study, we use μMap proximity labeling to identify how these mutations change the protein-protein interactions around p53. (B) Hotspot mutants are known to lead to altered gene expression through mislocalization at genomic loci and cofactor co-option at chromatin. μMap labeling reveals additional molecular activities of mutant p53, including RNA binding, cytoplasmic redistribution, and mitochondrial surface association, which collectively alter translational output and mitochondrial function.

The tumor suppressor protein p53 lies at the heart of cellular responses to stress. As a transcription factor, p53 maintains genomic integrity by activating a network of genes that coordinate DNA repair, cell-cycle arrest, senescence, and apoptosis.^1,2^ This central role in protecting against oncogenic transformation has earned it the title “guardian of the genome.” p53 is one of the most frequently altered genes in human cancer, being mutated in almost 50% of all tumors.^1,3^ Somatic mutations that disrupt its DNA-binding domain (DBD) disable its transcriptional programs, abolishing tumor suppression and, in many cases, conferring additional oncogenic properties (Figure 1).^4^

Among the hundreds of known p53 mutations, a majority falls into the DBD region; a small subset—often referred to as “hotspot” mutations—is recurrently enriched across tumor types and accounts for 28% of all p53 mutations.^3,5^ Their prevalence suggests that these mutants do more than simply lose transcriptional activity; rather, they may acquire distinct molecular functions that drive tumorigenesis.^3,4,6–9^ How these mutations acquire such oncogenic functions remains poorly understood.

Previous studies have used co-immunoprecipitation (coIP)^8,10^ zand chromatin immunoprecipitation (ChIP)^8^ to explore the interactions and genomic localization of mutant p53 (mut p53). These approaches have illuminated key binding partners and transcriptional programs but have critical limitations. CoIP methods capture stable protein-protein interactions but fail to preserve interactions mediated by nucleic acids or higher-order chromatin structures. Conversely, ChIP assays reveal DNA binding patterns but provide only indirect information about the dynamic and context-dependent complexes that define mut p53 function.

**Figure 2.**
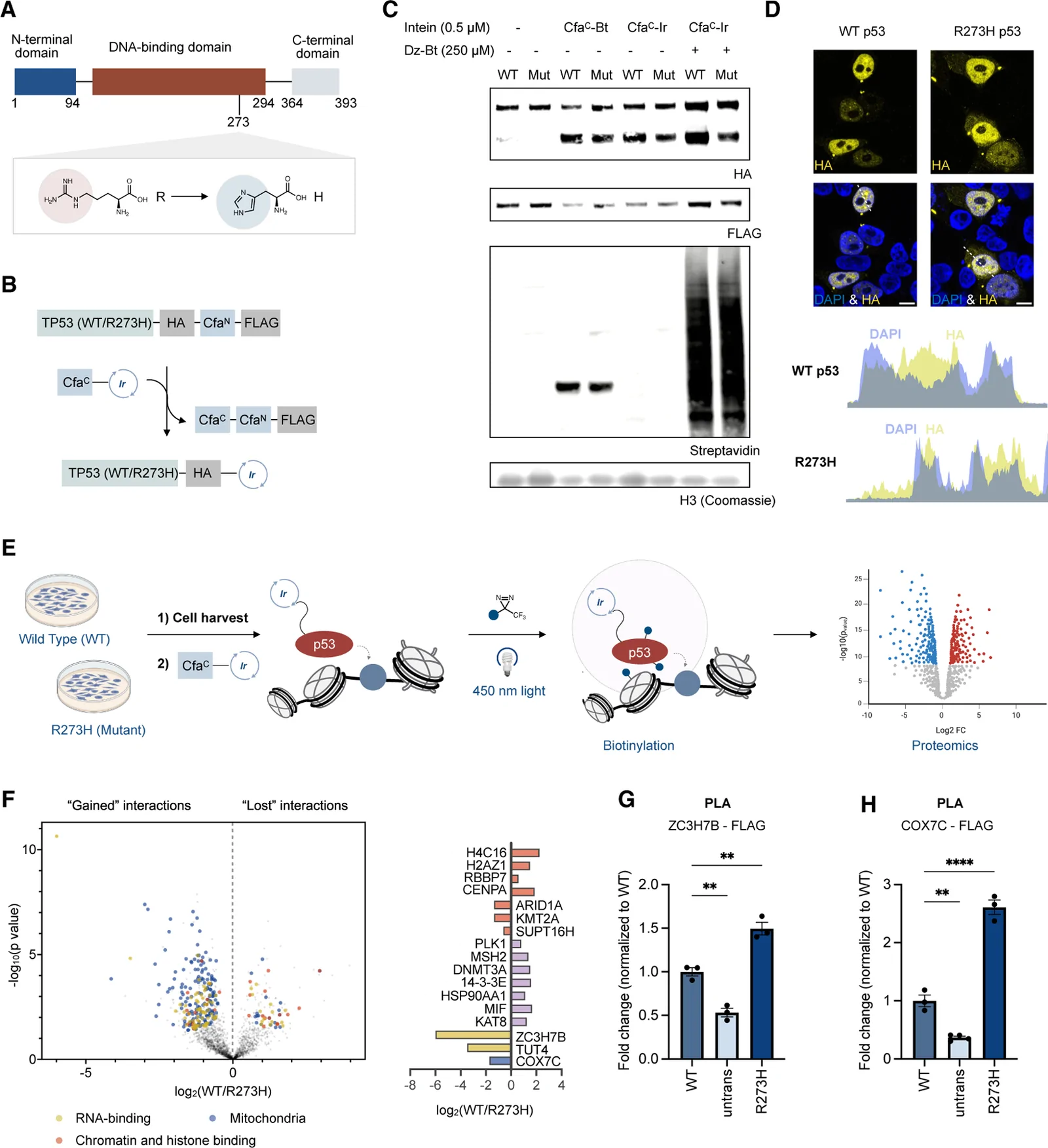
μMap proximity labeling uncovers the interactome of mutant p53 (A) Cartoon showing R273H mutation in the DBD of p53. (B) Scheme of intein splicing reaction. The N-terminal intein (Cfa^N^) is fused to p53, while the C-terminal intein (Cfa^C^) is conjugated to an Ir photocatalyst. Splicing leads to traceless conjugation of Ir onto p53. (C) Spliced product is only observed while treated with C-terminal intein (Cfa^C^). Streptavidin signal is only observed following treatment with Cfa^C^-Ir, Dz-Bt, and light. (D) Subcellular localization of WT p53 and R273H p53 in HEK293T cells. WT p53 exhibits a more concentrated nuclear localization. Scale bars, 10 μm. (E) Scheme of μMap proximity labeling experiment. HEK293T cells transiently expressing mutant or WT p53 were harvested, treated with intein-conjugated Ir (Cfa^C^-Ir), and labeled with 450 nm light. Labeled proteins were enriched and subjected to proteomic analysis. (F) Interactome shift caused by p53 R273H mutation. Left: a volcano plot derived from a two-sided *t* test comparing WT p53 interactors to R273H p53 interactors. FDR < 0.05. RNA-binding, chromatin and histone binding, and mitochondrial proteins are annotated. Right: enrichment of selected proteins in the dataset. (G) Proximity ligation assay (PLA) confirming the interaction between p53 and ZC3H7B (*n* = 3, one-way ANOVA test followed by post hoc Dunnett’s multiple comparisons comparing to untransfected control. **p < 0.01). PLA puncta per field of view were quantified for analysis (mean±SEM). (H) PLA confirming the interaction between p53 and COX7C (*n* > 3, one-way ANOVA test followed by post hoc Dunnett’s multiple comparisons comparing to untransfected control. **p < 0.01, ****p < 0.0001). PLA puncta per field of view were quantified for analysis.

To overcome these challenges, proximity labeling (PL) has emerged as a powerful approach to map molecular interactions in their native environments.^11–13^ PL methods can capture transient, weak, or nucleic acid-scaffolded interactions that are otherwise inaccessible to traditional biochemical techniques. Building on these advances, we sought to apply μMap,^14^—a photocatalytic PL strategy developed for high-precision interactomics—to study how hotspot mutations reshape the molecular landscape surrounding p53. μMap operates through the short-lived generation of carbenes that label proximal biomolecules within nanometer-scale distances,^15^ providing an exceptionally sensitive probe of local molecular architecture. This property makes μMap ideally suited to distinguishing subtle structural or conformational differences between wild-type and mutant proteins (Figures 1, 2A, and 2E).

To bring μMap labeling directly to p53 in its native nuclear context, we leveraged a split-intein delivery system recently developed in our laboratory^16,17^ (Figures 2B and 2E). Compared to conventional PL approaches that require bulky tag insertion such as APEX, TurboID, or HaloTag (~28–35 kDa), the split-intein-delivered μMap photocatalyst (~12 kDa) minimizes perturbation of p53 tetramerization and thus is well-suited for studying the p53 interactome.

In this approach, p53 is expressed as a fusion with one-half of a split intein, flanked by small epitope tags (Figure 2B). Upon cell permeabilization, the complementary intein fragment bearing a pendent iridium photocatalyst is introduced. Rapid intein splicing seamlessly incorporates the photocatalyst onto p53, enabling precise labeling of its immediate molecular environment without disrupting native cellular architecture (Figure 2E). This system allows μMap profiling in permeabilized cells with intact nuclei—conditions particularly suited to studying a transcription factor such as p53, whose function is deeply intertwined with chromatin structure and RNA interactions. We applied this platform to map how five hotspot p53 mutations reshape the protein interaction landscape.

## RESULTS

### Method development

We initiated our study by expressing plasmids containing either p53-HA-Cfa^N^-FLAG or mut R273H in HEK293T cells, which are insensitive to p53 expression. We used the DNA-binding domain mutation, R273H, as an exemplar due to the availability of prior gain-of-function studies and its abundance in a variety of tumor types (Figure 2A). We validated intein-splicing of our constructs using Cfa^C^-biotin and Cfa^C^-Ir (Figure 2C). Incubation with Cfa^C^-biotin for 30 min at 37°C led to a shift in the construct mass as measured by western blot, in addition to a corresponding streptavidin signal at a mass indicative of full-length p53, consistent with extein loss and incorporation of biotin at the C-terminus of p53. Cells treated with Cfa^C^-Ir were then irradiated at 450 nm for 60 s in the presence of diazirine-PEG3-biotin (Dz-Bt) to induce protein labeling. Streptavidin western blot demonstrated significant biotinylation only when all three conditions were simultaneously present—the transfected construct, Dz-Bt, and light exposure—confirming the specificity of the μMap labeling reaction. This specificity was further validated in permeabilized cells by immunofluorescence (Figure S1A). We next demonstrated localization of wild-type and mutant construct R273H via confocal microscopy, showing substantial nuclear localization in both cases, with some non-nuclear signal observed in the mutant (Figure 2D).

We first validated that our construct produces a high-quality p53 interactome by comparing wild-type p53 (WT p53) to an untransfected control. Each condition was performed with three biological replicates, each with two technical replicates per biological replicate, to account for experimental variability. For hit determination, we defined significance thresholds of false discovery rate (FDR) < 0.05. Compartmental analysis of the identified proteins showed that 51% of all IDs in the enriched proteome of p53 were localized to the nucleus, 21% to the mitochondria, and 28% to other cellular compartments. This distribution reflects the compartmental annotations of proximal neighbors rather than the localization of p53 itself, as PL captures transient and sub-stoichiometric interactions that extend beyond the predominant localization of the bait protein. Gratifyingly, we observed strong enrichment of p53 itself and several canonical binding proteins (*EP300*,^18,19^ *KAT2A*,^20^ *PML*,^21,22^ and *ATR*^23^) among our top hits, with enriched GO terms relevant to p53 function (Figures S1B and S1C).

### p53 R273H blocks chromatin binding and associated interactions

Upon successful PL of p53, we proceeded to generate interactomics data for WT and R273H p53 via label-free chemoproteomics to determine if we could delineate between interactomes of proteins that differ by only a single amino acid residue.

Analysis via volcano plot revealed 112 proteins significantly enriched with WT p53 and 337 significantly enriched with the mutant (Figure 2F). Throughout, we use “‘lost’ interactions” to describe interactions enriched with WT p53 but reduced in the mutant and “‘gained’ interactions” to describe interactions enriched with the mutant relative to wild type; these terms refer to the interaction comparison, not to loss or gain of DNA binding, which is shared by all mutants studied. GO analysis of the “lost” interaction hits showed significant enrichment of chromosome organization, mitotic cell cycle, and regulation of DNA and RNA metabolism—all key functions of WT p53,^2,24–26^ consistent with a loss of DNA-binding capacity and chromatin localization (Figures S1C and S1D). This was supported by enrichment of 15 proteins related to histones, histone binding, and chromatin organization (e.g., H4C16, H2AZ1, RBBP7, and CENPA) in the wild-type interactome (Figure 2F). We also observed a robust enrichment of proteins associated with DNA-related processes closely tied to p53 function, including PLK1,^27^ MSH2,^28^ and DNMT3A.^29^ In addition to chromatin-localized proteins, several known proteins that regulate p53 function through direct interactions were enriched in the wild-type samples. For example, the 14-3-3ε interacts with the disordered C-terminal domain of p53 and promotes tetramerization and transcriptional activity.^30^ It has been reported that HSP90AA1 binds to p53 and stabilizes the DNA-binding domain.^31^ Likewise, binding to MIF, which regulates p53 transcriptional activity in response to immune challenge,^32^ is diminished in the R273H samples. In addition to proteins that directly regulate p53 stability and function, we identified KAT8, an acetyltransferase, which acetylates K120 of p53^33^ and mediates p53-based transcription-dependent apoptosis.

### p53 R273H displays “gained” interactions

Gene Ontology (GO) analysis of the “gained” interactions of p53 (R273H) revealed significant enrichment of terms related to RNA processing (e.g., ZC3H7B and TUT4), mitochondrial organization (COX7C), translation, and aerobic respiration, pointing to additional functions of the mut p53 (Figures 2F and S1D). Additionally, we observed a small number of “gained” interactions with chromatin-bound proteins (ARID1A, KMT2A, and SUPT16H), although these were substantially fewer than proteins associated with RNA or mitochondrial function, consistent with a mechanism where R273H reduces affinity for chromatin and redirects p53 away from its canonical transcriptional interactome toward other functions (Figure 2F). To ensure the validity of our proteomic hits, we performed proximity ligation assays (PLAs)^34^, to measure the abundance of two of our most enriched interactions (ZC3H7B and COX7C) in fixed cells. Using this technique, we measured a 1.5-fold increase in proximity between R273H and ZC3H7B and a 2.5-fold increase in proximity with COX7C when compared to WT p53 (Figures 2G and 2H; Figure S1F).

Importantly, IP-MS of the same constructs was not instructive, with few differential interactions observed, suggesting that cellular localization, rather than altered binding surfaces, drives differential protein-protein interactions and providing further evidence that IP-MS protocols may be confounded by post-lysis changes to protein interactomes (Figure S1E). Furthermore, comparison of enriched genes to the bulk proteome showed that enriched proteins were a result of proximity rather than changes in protein levels (Figure S1G).

### p53 hotspot mutations display different localization and binding affinities

The recurrent enrichment of a small number of hotspot mutations across cancer types suggests they may share a conserved gain-of-function mechanism beyond simple loss of DNA binding. Having identified RNA processing and mitochondrial interactions as “gained” interaction features in R273H, we next asked whether these represent a general class effect of DBD mutations or an idiosyncrasy of a single substitution—a distinction critical to the broader relevance of our findings. To address this, we extended our PL platform to a panel of four additional hotspot mutations (R248Q, R282W, G245S, and R249S), representing both contact and structural mutation classes (Figure 1A).

**Figure 3.**
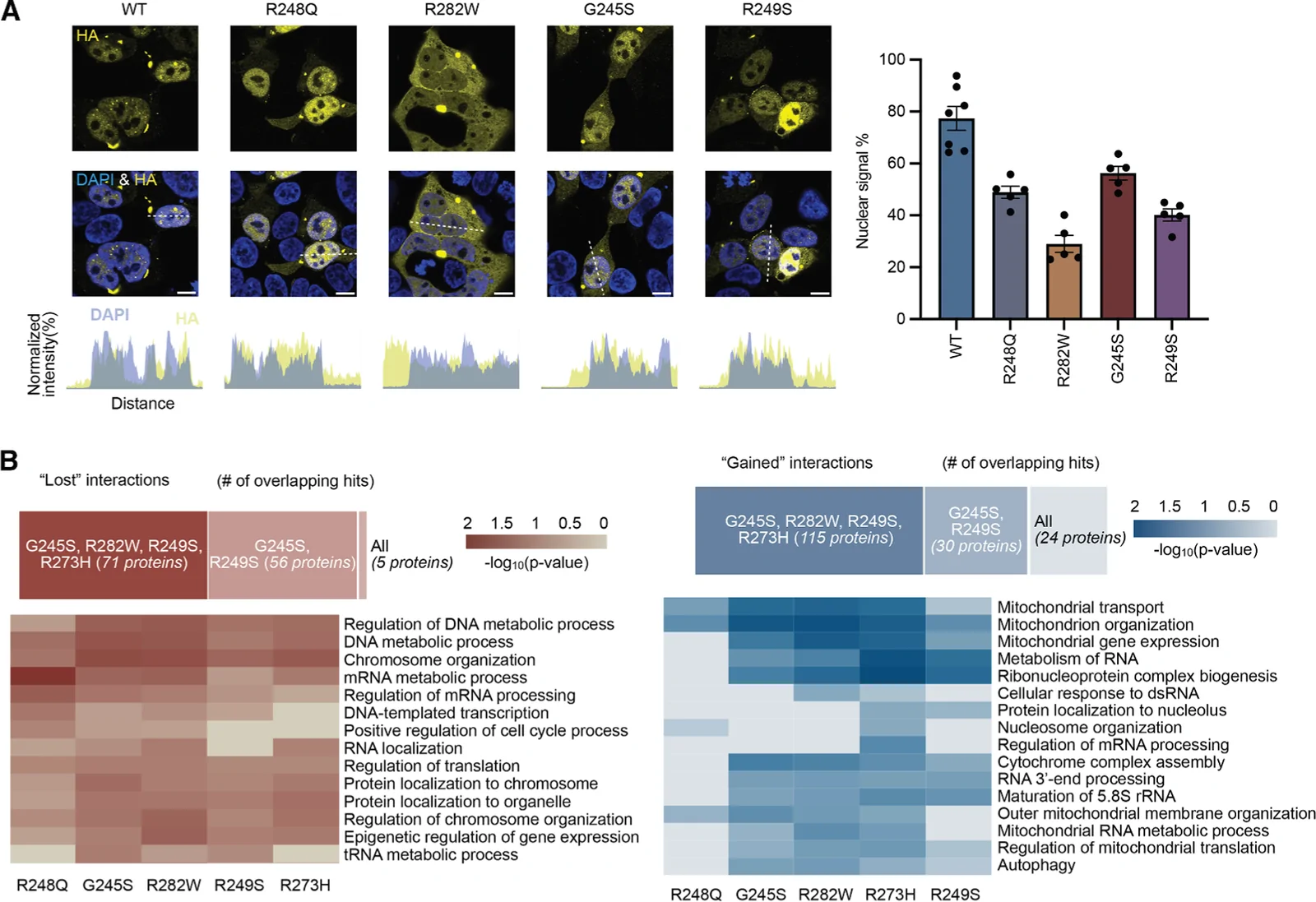
Unraveling functional changes in p53 with known hotspot mutations (A) Subcellular localization of WT p53 and mut p53 in HEK293T cells. WT p53 is more highly localized in the nucleus than any mutant. Scale bars, 10 μm (mean±SEM). (B) Each mutant displays a unique interactome although some overlap is observed in “lost” interactions, “gained” interactions, and enriched gene ontologies. Hierarchical cluster analysis was performed based on the enrichment score of GO terms.

Prior to chemoproteomic characterization, we confirmed the efficiency of construct expression and splicing with Cfa^C^-biotin, demonstrating effective intein splicing across all constructs tested (Figure S2A). Following this, we determined the cellular localization of each construct through cellular fractionation and confocal microscopy (Figure 3A). These data clearly show substantial differences between p53 localization depending on the mutation under study, with all mutants exhibiting higher cytoplasmic localization (Figure S2B).

We next performed our PL experiment with these p53 hotspot mutants to determine shared gain-of-function roles among mutations. Their shared abundance across tumors of different line-ages may suggest a conserved set of interactors that drive their enrichment in cancer.^5^ Broadly, we observe that the degree of interactome change is dependent on the identity of the mutant, and, consistent with their localization, that not all mutations bear identical interactomes. Hierarchical clustering of the data shows that the serine mutants G245S and R249S that alter p53 structure (“structural mutations”^35^) bear the most similarity and that R273H and R248Q, which block DNA binding directly (“contact mutations”^35^), also cluster together^3^ (Figures 3B and S2D). Of the mutations tested, R248Q exhibited the weakest effect on the interactome, with 106 differentially enriched proteins, while R282W showed the strongest, with 1,168 differentially enriched proteins (FDR = 0.05; Figure S2C).

GO analysis of the “gained” interaction terms across all five mutants showed consistent enrichment for mitochondrial localization (Figure 3B). RNA processing was also significantly enriched in all mutants except R248Q. Together, these data suggest that somatic mutations in the p53 DNA-binding domain promote mislocalization of the protein into the cytosol and mitochondria, enabling aberrant interactions with proteins involved in mitochondrial function and RNA processing. While each mutant displays unique features, several share substantial overlap in their biochemical interactions (Figure 3B). Based on these patterns, we selected R273H and G245S for further investigation, as they broadly represent the two major clusters and functional classes of hotspot mutations. Prior to follow-up studies, we generated stable cell lines containing either WT p53, R273H p53, or G245S p53 and measured the impact that the mutants had on the transcriptome through RNA-seq analysis. Pathway analysis showed that when compared to cells expressing WT p53, both mutants effectively suppressed p53-based transcriptional activity, suggesting that they engage in dominant-negative transcriptional behavior (Figures S2E and S2F).

### Mut p53 acquires interactions with RNA-binding proteins and shifts toward the cytoplasm

**Figure 4.**
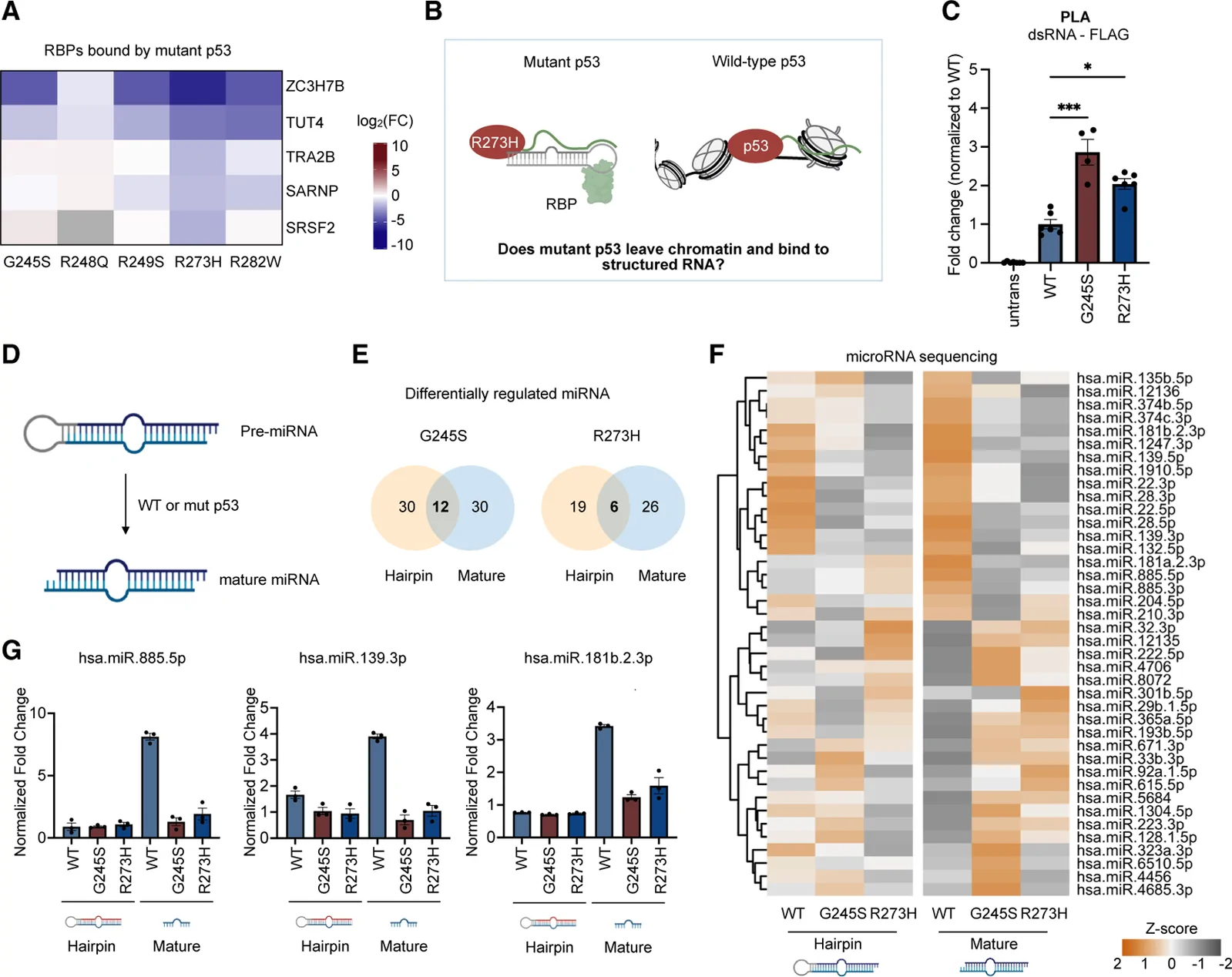
Mutant p53 regulates miRNA biogenesis (A) A heatmap of RBP enrichment score across different mutations. (B) Cartoon illustrating how mutant p53 RNA binding might alter localization. (C) PLA confirmation of interaction between p53 and *ds-*RNA (*n* ≥ 4, one-way ANOVA test followed by post hoc Dunnett’s multiple comparisons comparing to untransfected control. *p < 0.05, ****p < 0.0001). PLA puncta per field of view were quantified for analysis (mean±SEM). (D) A diagram showing p53 participation in miRNA processing. (E) Venn diagram showing differentially regulated hairpin and mature miRNA in mutant p53 (normalized to WT p53). (F) Heatmap showing *Z* score of miRNAs that are differentially regulated on the mature RNA level but not regulated on the hairpin level in both mutants. *Z* score is calculated within each miRNA in both hairpin and mature RNA. (G) Individual bar plots showing selected miRNAs relevant to tumor suppression that are regulated at the hairpin level. *n* = 3 biological replicates for each condition (mean±SEM).

Of the “gained” interaction proteins associated with metabolism of RNA, we consistently enriched ZC3H7B, an RNA-binding protein associated with miRNA biogenesis,^36,37^ in addition to several other RNA processing proteins (TUT4, TRA2B, SARNP, and SRSF2) (Figure 4A). As many of these proteins were enriched regardless of the identity of the mutant, we questioned whether, in the absence of a high-affinity DNA-binding interaction, mut p53 exhibits altered localization and a preference for binding to RNA and associated RNA-binding proteins.

To test this, we performed PLA using antibodies against FLAG (WT, R273H, and G245S) and an antibody that recognizes double-stranded and structured^38^ regions of RNA^39^ (*ds*-RNA K1) to quantify interactions between p53 and structured regions of RNA (Figure 4B and 4C; Figure S3). We observed a 2.0-fold increase in interaction with *ds*-RNA for R273H and a 2.9-fold increase with G245S compared to WT p53, suggesting enhanced affinity of the mutant proteins for regions of double-stranded RNA (>40 bp). Broadly, RNA-binding capacity by PLA was consistent with RNA-binding GO terms derived from PL (Figure 3B). This result further supports the proposed model that mut p53 may engage in non-canonical regulatory mechanisms involving RNA interactions rather than traditional DNA binding. These assays establish RNA-dependent proximity to structured RNA without defining the full set of RNA species engaged, which is further explored through CLIP-seq below.

Furthermore, as these microscopy-based assays report co-localization rather than direct binding, we cannot at this stage discriminate between direct interactions and interactions scaffolded by either proteins or RNA. However, as conventional Co-IP experiments have uniformly failed to identify ZC3H7B and TUT4 as mut p53 binders, we propose that the primary binding is to RNA, which facilitates proximity to RNA processing proteins.

It has been widely established that p53 interacts with the Drosha complex and assists in miRNA processing, while mut p53 has been reported to interact with and regulate biogenesis factors involved in miRNA biogenesis.^40–42^ As our PL dataset has captured mut p53 interacting with miRNA processing factors (ZC3H7B^37^ and TUT4^43^), we sought to better assess how these interactions influence miRNA precursor processing and maturation. We performed miRNA-seq on cells stably expressing WT p53, R273H-p53, and G245S-p53 (Figures S4A and S4B). To differentiate between miRNAs that are regulated by the transcriptional function or through altered localization and binding of p53, we filtered the data to identify RNAs that are selectively regulated at the mature transcript but remain unchanged at the precursor level (Figure 4D). For G245S, we observed 42 differentially regulated miRNAs, of which 30 were only regulated at the mature miRNA level (*p*_adj_ < 0.1), while for R273H, 26 out of 32 transcripts were regulated at the mature miRNA level, consistent with mut p53-driven disruption of miRNA processing^40,44^ (Figures 4E and 4F). Of specific interest were a set of tumor-suppressing miRNAs (miR-28,^45^ miR-139,^46^ miR-181,^47,48^ miR-204,^49^ miR-671,^50^ and miR-885^51,52^) that were significantly regulated only at the mature form, suggesting that mutp53 obstructs their processing either through direct binding to precursor hairpins or indirectly through miRNA-processing factors identified in our PL dataset (Figures 4F and 4G). Notably, we identified significant downregulation of both miR-582–5p and miR-582–3p in R273H-expressing cells relative to WT p53, a finding that mirrors an independent report of miR-582 suppression by endogenous mut p53 R273H in SW480 colon cancer cells.^41^

**Figure 5.**
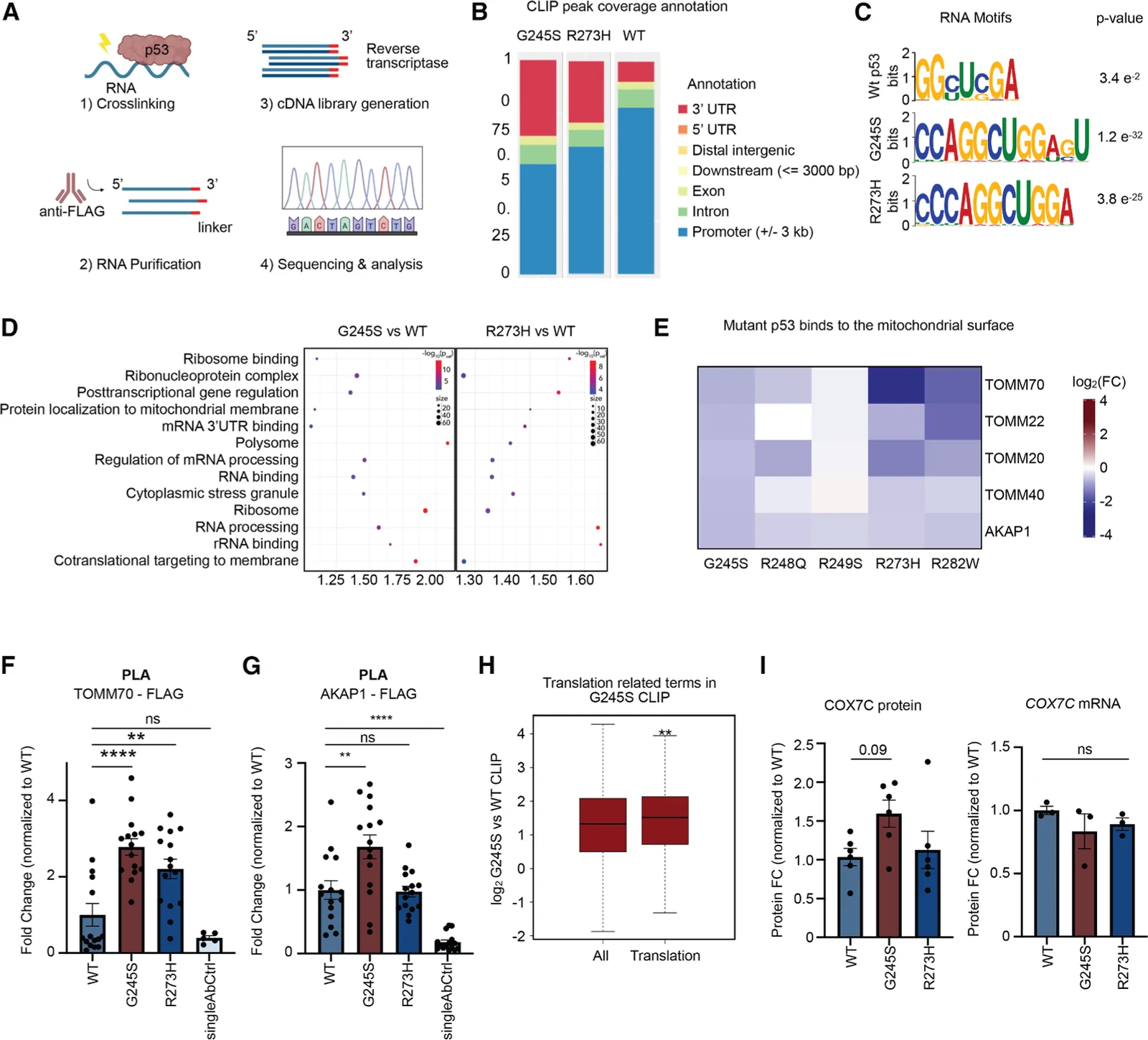
Mutant p53 binds RNA at mitochondrial surface (A) Schematic of CLIP-seq workflow. (B) CLIP-seq peak coverage in genomic regions. (C) A consensus RNA-binding motif containing a common GGCUGG sequence is shared between wild-type (WT) and mutant p53. This motif represents the most enriched sequence in RNAs bound by WT and G245S p53 and the second most enriched motif in R273H p53. Motifs were identified using STREME analysis (MEME Suite) from peaks present in both biological replicates. (D) Normalized Enrichment Score (NES) from Gene Set Enrichment Analysis (GSEA) of CLIP-seq peaks. Gene sets commonly enriched in both mutant versus WT conditions are shown. (E) A heatmap showing enriched proteins that localize to the mitochondrial outer membrane identified through μMap PL across different mutations. (F) PLA confirmation of interaction between p53 and TOMM70 (*n* = 15, one-way ANOVA test followed by post hoc Dunnett’s multiple comparisons comparing to WT control. **p < 0.01, ****p < 0.0001; ns, not significant). PLA puncta per field of view were quantified for analysis (mean ± SEM). (G) PLA confirmation of interaction between p53 and AKAP1 (*n* = 15, one-way ANOVA test followed by post hoc Dunnett’s multiple comparisons comparing to WT control. **p < 0.01, ****p < 0.0001; ns, not significant). PLA puncta per field of view were quantified for analysis (mean ± SEM). (H) Among G245S-bound transcripts, RNAs associated with translation highly enriched. Translation-related proteins were retrieved from MSigDB (GOBP: Translation; GO:0006412). Statistical significance was calculated using the Wilcoxon signed-rank test. **p < 0.01. (I) Mut p53-bound COX7C has increased expression but no upregulation at the transcript level. Left: COX7c protein expression level in global proteomics (n = 6, mean ± SEM, one-way ANOVA test followed by post hoc Dunnett’s multiple comparisons comparing to WT control; *, p < 0.05). Right: COX7C transcript level addressed by RT-qPCR (n = 3, mean ± SEM, one-way ANOVA test followed by post hoc Dunnett’s multiple comparisons comparing to WT control; ns, not significant).

The selective regulation of mature, but not precursor, miRNAs suggests that mut p53 disrupts RNA processing rather than transcriptional regulation. Together with our previous observations of increased *ds*-RNA interactions and enhanced cytosolic localization, these findings point to a broader alteration in RNA-binding behavior. To directly examine this, we performed CLIP-seq on WT p53, R273H, and G245S (Figure 5A) to define their RNA-binding profiles genome-wide. We observed significantly increased RNA-binding in both mutants, in line with our PL and PLA datasets (Figure S5A). Each mutant bears a distinct binding profile compared to wild-type as visualized by PCA, with good clustering among replicates (Figure S5B). DESeq2 analysis showed a dramatic difference in transcript identity, with WT p53 binding to intronic RNA and promoter regions, consistent with its nuclear localization. Both mutants demonstrated preferential binding to 3′UTRs that contain both structured and unstructured regions, with the most dramatic effect observed in G245S (Figure 5B), consistent with GO terms (RNA 3′-end processing) observed in our proximity proteomics (Figure 3B). Furthermore, motif analysis showed that wild-type and mutants shared enrichment for a common consensus sequence centered on GGCUGG, suggesting that p53 has an intrinsic RNA-binding preference (Figure 5C).

Strikingly, this GGCUGG-centered consensus corresponds to a segment of the antisense Alu element—the most abundant interspersed repeat in the human genome and one that is densely embedded within 3′UTRs.^53,54^ Because Alu repeats are inserted in both orientations, inverted Alu pairs fold back on themselves to form extended *ds*-RNA structures detectable by the K1 antibody,^39,55^ providing a unifying, structural explanation for our earlier observations: the increased proximity of mut p53 to *ds*-RNA detected with the K1 antibody (Figure 4C) and the pronounced enrichment of mut p53 CLIP peaks within 3′UTRs (Figure 5B). We therefore interpret this motif not as a sequence-specific recognition element but as a marker of Alu-derived structured/double-stranded RNA, consistent with a model in which loss of DNA binding shifts p53 toward the recognition of RNA structure rather than a defined linear sequence. The further enrichment of the motif in the mutants (Figure 5C) indicates that the disruption of the DNA-binding domain heightens this intrinsic, Alu/structure-directed RNA preference (see Figure S5C for additional enriched motifs). Gene set enrichment analysis (GSEA) of bound RNAs across both mutants showed binding to ribosomal transcripts and those associated with co-translational targeting to organelle membranes, implying that mut p53 binds to 3′UTRs of RNAs translated at the ER^56^ and mitochondria^57^ (Figure 5D). Re-analysis of our PL data supported this with enrichment of mitochondrial outer membrane proteins TOMM70, TOMM22, TOMM20, and TOMM40, in addition to co-translational scaffold protein AKAP1 (Figure 5E), which was previously found to be localized to the mitochondrial surface.^58^ Consistently, PLAs confirmed mutant-specific interactions between p53 and mitochondrial components TOMM70 (Figures 5F and S5D) as well as AKAP1 (Figures 5G and S5D), supporting a model in which mut p53 engages RNA and protein partners at the mitochondrial surface (Figure S5D).

We next cross-analyzed our CLIP and PL datasets to identify mitochondrial proteins whose transcripts are bound by mut p53 and whose encoded proteins co-localize with mut p53, potentially indicating a role for mut p53 in co-translational targeting to the mitochondria. Among these, we identified that COX7C, an essential component of the electron transport chain,^59^ was bound more by mut p53 at both the RNA and protein levels (Figure S5F). RT-qPCR analysis showed that the *COX7C* transcript remained unchanged in all conditions, while the presence of mut p53 increased its protein levels (as shown by global proteomics analysis) (Figure 5I). We also noted several global trends that were supportive of our hypothesis. Correlation of the global proteomic and CLIP datasets revealed that translation-associated proteins not only showed increased abundance in the mutant proteome but also had their mRNAs bound by the G245S mutant (Figure 5H). Furthermore, mitochondrial proteins, in addition to being enriched in the PL dataset of mut p53, were also enriched in the global proteomes for both mutants studied (Figure S5E).

**Figure 6.**
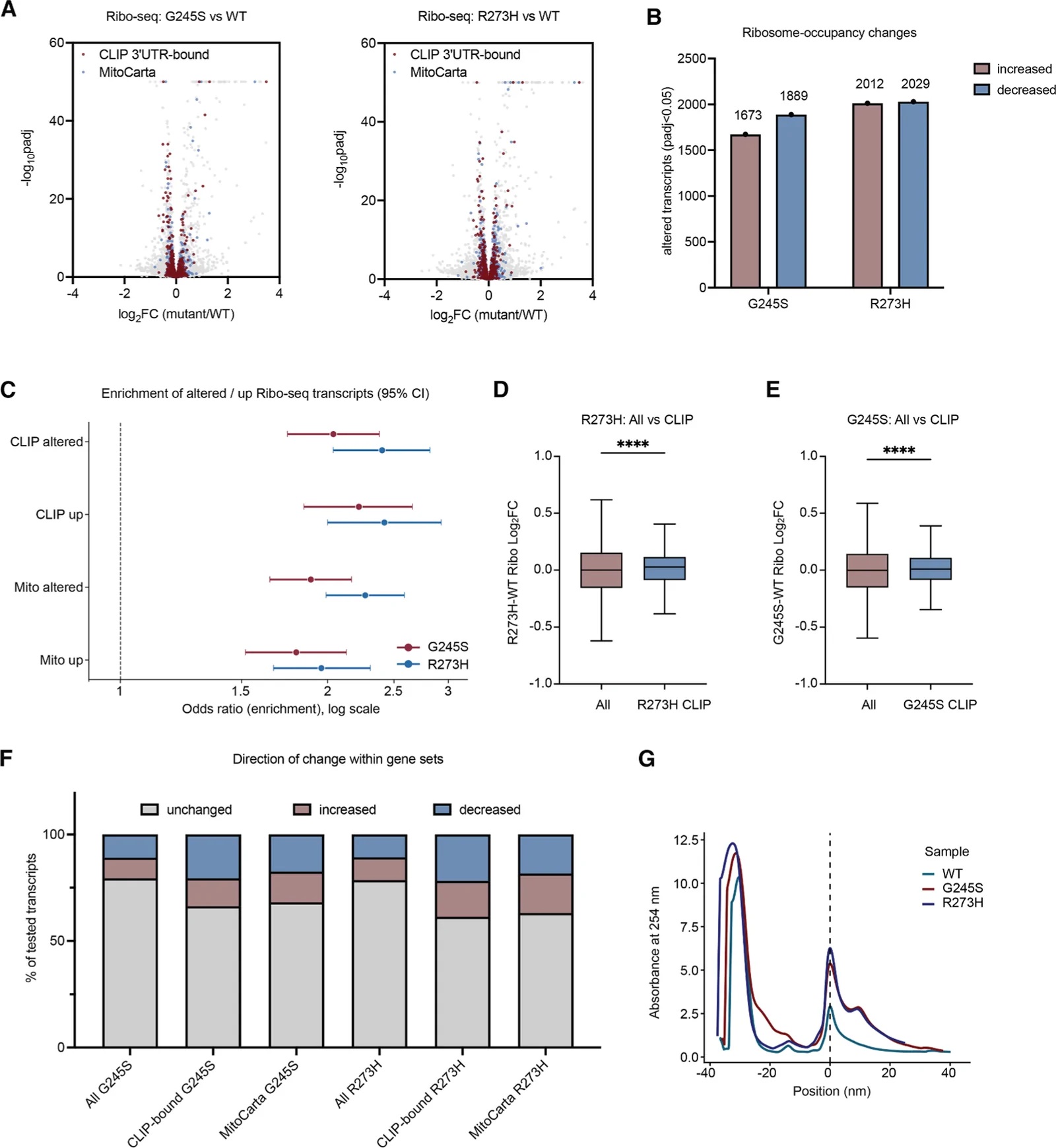
Mutant p53 directs ribosome occupancy toward its bound and mitochondrial mRNAs (A) Volcano plots of differential ribosome occupancy (Ribo-seq) in G245S- and R273H-expressing cells relative to WT p53 (log_2_ fold change, mut/WT; Wald test, Benjamini-Hochberg-adjusted *p*). Transcripts whose 3′UTRs are bound by mut p53 (CLIP, dark red) and mitochondrial transcripts (MitoCarta3.0, blue) are highlighted against all other quantified transcripts (gray). (B) Number of transcripts with significantly altered ribosome occupancy (*p*_adj_ <0.05), separated by direction (increased or decreased in the mutant). (C) Enrichment of CLIP 3′UTR-bound and mitochondrial (MitoCarta3.0) transcripts among all altered transcripts (“altered”) and among transcripts with increased occupancy (“up”), expressed as odds ratios (ORs) with 95% confidence intervals (Fisher’s exact test); dashed line, odds ratio (OR) = 1. (D) and (E) Distribution of Ribo-seq fold change (mut – WT, log_2_) for all quantified transcripts (“All”) versus mut-p53 CLIP 3′UTR-bound transcripts, for R273H (D) and G245S (E). Box, interquartile range and median; whiskers, Tukey. ****p < 0.0001, two-sample Kolmogorov-Smirnov test. (F) Proportion of transcripts with increased, decreased, or unchanged ribosome occupancy within all quantified transcripts, CLIP 3′UTR-bound transcripts, and mitochondrial transcripts, for each mutant. (G) Monosome profiles (absorbance at 254 nm across sucrose density gradients) of cells expressing WT, G245S, or R273H p53; the dashed line marks the 80S/polysome boundary.

To directly test whether the 3′UTR-binding activity of mut p53 identified by CLIP and the proximity to ribosomal and mitochondrial components established by our PL and confirmed by PLA have functional consequences for translation, we performed ribosome profiling (Ribo-seq) in stable HEK293T cell lines expressing WT, G245S, or R273H p53. As Ribo-seq measures ribosome occupancy at single-transcript resolution genome-wide, it allows us to ask whether the transcripts bound by mut p53 at their 3′UTRs show altered translational output in mutant-expressing cells. Results showed that mut p53 reshapes the translational landscape, significantly altering ribosome occupancy on 3,562 transcripts in G245S and 4,041 in R273H cells (*p*_adj_ < 0.05; Figures 6A and 6B). These altered transcripts were strongly enriched for the 3′UTR-bound mRNAs identified by CLIP and for mitochondrial transcripts (MitoCarta3.0) (odds ratios: 2.0–2.4 and 1.9–2.3, respectively; all *p* < 0.0001, Fisher’s exact test; Figure 6C), and the same enrichment held when we considered only the transcripts whose occupancy increased in the mutant (odds ratios: 2.2–2.4 and 1.8–2.0; Figure 6C). Consistently, the CLIP 3′UTR-bound transcripts as a group showed a fold-change distribution shifted toward increased occupancy relative to the transcriptome as a whole (*p* < 0.0001, Kolmogorov-Smirnov test; Figures 6D and 6E), and increased-occupancy transcripts comprised a larger fraction of the bound and mitochondrial sets than of all transcripts (Figure 6F). At the global level, MNase-digested lysates prepared from matched cell numbers and equivalent RNA inputs showed increased 80S monosome signal in both mut p53-expressing lines relative to wild-type cells (Figure 6G). Because these profiles were generated after nuclease digestion and monosome isolation rather than conventional polysome profiling, these data indicate an increased abundance of ribosome-protected 80S complexes under matched input conditions. Together with the Ribo-seq analyses, these functional data support a model in which the 3′UTR-binding activity of mut p53 enhances ribosome occupancy and translational output of its bound transcripts, including mitochondrial mRNAs, linking mut p53 RNA-binding behavior to the mitochondrial phenotypes described below.

Taken together, these data point to a coordinated shift in RNA and protein interactions that positions mut p53 at the interface of mitochondrial and translation regulation, hinting at broader consequences for cellular metabolism.

### Mut p53 leads to changes in mitochondrial function

Across our PL, CLIP, and Ribo-seq datasets, mitochondrial proteins and mitochondrially targeted mRNAs emerged consistently as shared gained interactions among hotspot mutants. As WT p53 is known to translocate to the mitochondrial membrane to promote apoptosis,^57,60^ we hypothesized that loss of DNA-binding redirects mut p53 toward altered mitochondrial function. We therefore asked whether these molecular interactions have functional consequences for mitochondrial physiology, measuring mitochondrial mass, membrane potential, ROS, and respiratory function as orthogonal readouts of mitochondrial health and metabolic capacity.

**Figure 7.**
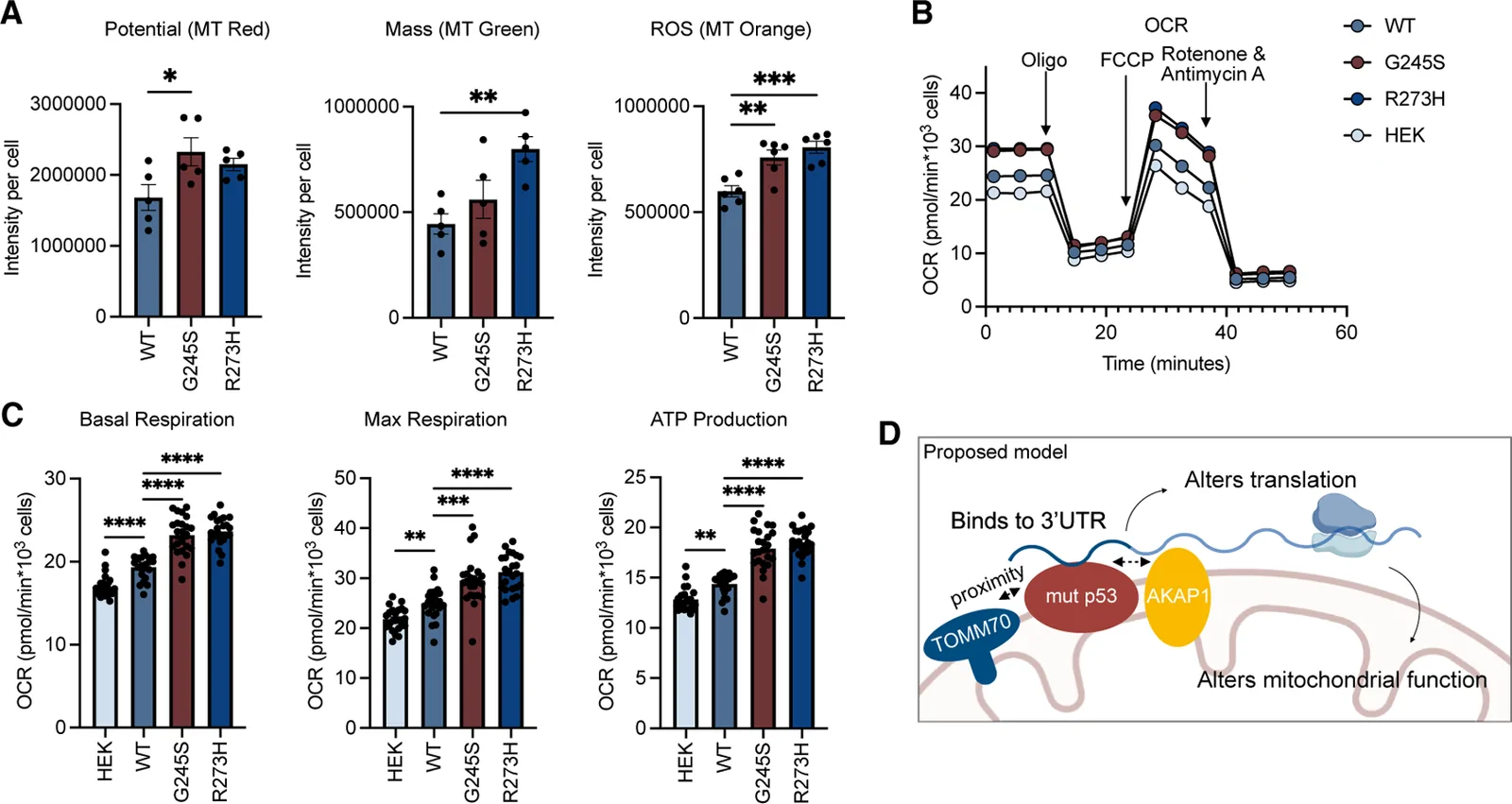
Mutant p53 alters mitochondrial function (A) Expression of mutant p53 leads to changes in mitochondrial mass, membrane potential, and ROS level (*n* ≥ 5, one-way ANOVA test followed by post hoc Dunnett’s multiple comparisons comparing to WT control. *p < 0.05, **p < 0.01, ***p < 0.001). Mitotracker intensity per field of view was quantified for analysis (mean ± SEM). (B) Mitochondrial respiration was measured by Seahorse assay. Data are presented as mean ± SEM of *n* = 23 technical replicates. Experiment was performed in biological duplicate; a representative experiment is shown. (C) Basal respiration, max respiration, and ATP production were altered due to mutant p53 expression in cells (mean ± SEM, n = 23 technical replicates, one-way ANOVA test followed by post hoc Dunnett’s multiple comparisons comparing to WT control; **p<0.01, ***p<0.001, ****p<0.0001). (D) A cartoon showing a model of mutant p53 localizes on mitochondrial outer membrane and binds to RNA and AKAP1.

To quantify the impact of mut p53 on mitochondrial function, we measured mitochondrial mass, membrane potential, and reactive oxygen species (ROS) via MitoTracker microscopy assays. We found that both R273H- and G245S-p53 showed increased mitochondrial mass (MitoGreen) and increased mitochondrial potential (MitoRed) relative to WT p53, with statistical significance reached for mass in R273H-expressing cells and for potential in G245S-expressing cells, suggesting that mitochondrially localized mut p53 has a negative functional impact on mitochondrial health (Figures 7A and S6). We also investigated cellular ROS and respiration. MitoTracker Orange, which requires ROS to activate it prior to mitochondrial accumulation, showed a 1.2- to 1.3-fold increase in both mutants compared to wild-type (Figures 7A and S6). These data were confirmed by Seahorse analysis, which showed a significant increase in basal and maximal respiration and ATP production in cells stably expressing mut p53 when compared to WT p53 (Figures 7B and 7C). Thus, mut p53’s mislocalization and RNA-binding properties extend beyond molecular interactions and are associated with altered mitochondrial physiology. Figure 7D integrates our PL, CLIP, and localization data into a proposed model of how mut p53 engages RNA and protein partners at the mitochondrial surface.

### Mut p53 RNA regulation generalizes across human tumors

Although our biochemical and functional data were obtained in an engineered HEK293T system, we sought to test whether the RNA-centric behavior of mut p53 is recapitulated in human cancers. We analyzed mature-miRNA, mRNA, mutation, protein, and tumor-purity data for 10,323 tumors from The Cancer Genome Atlas (TCGA) Pan-Cancer Atlas, stratifying TP53 by allele class and modeling each feature against TP53 status with cancer type as a covariate to control for tissue of origin. The tumor-suppressor miRNAs that we found to be selectively processed in our cells were lower in TP53-mutant tumors: mature miR-139–5p, miR-139–3p, and miR-204–5p were each significantly reduced relative to WT TP53 (miR-139–5p: β = −0.23, *q* ≈ 1 × 10^−12^; miR-139–3p: β = −0.196, *q* ≈ 3.5 × 10^−9^; miR-204–5p: β = −0.21, *q* ≈ 2 × 10^−4^; Figure S7A). Consistent with a model in which loss of DNA binding—rather than any single substitution—drives this behavior, the reduction was seen across the entire class of DNA-binding-domain missense mutants rather than at individual hotspots, and was also present in truncating/null tumors, indicating a contribution from loss of WT p53 function. For miR-139, however, the reduction additionally scaled with mut p53 protein abundance and was specific to mutant, not wild-type, tumors (mutant × p53 interaction β = −0.22, *p* < 1 × 10^−4^), persisting after adjustment for tumor purity (Figure S7B)—consistent with an additional, mut p53-dependent component superimposed on loss of function. These observations that point toward effects shared across all DNA-binding missense mutants are consistent with our primary PL data, where increased association to RNA-binding proteins was shared among the hotspot mutants tested.

We next connected our CLIP data to these tumors. Transcripts bound by mut p53 at their 3′UTRs were enriched for the GGCUGG-centered consensus relative to non-bound genes (28% vs. 20%; odds ratio = 1.59, *p* = 2.5 × 10^−8^) (Figure S7C), and transcripts whose 3′UTRs contained this motif were modestly but dose-dependently elevated in TP53-mutant tumors, independent of 3′UTR length and GC content (β = +0.010, *p* = 0.017; Figure S7D). Notably, the transcripts that mut p53 bound at their 3′UTRs in our CLIP experiments were coordinately higher in TP53-mutant tumors (median +0.026 log_2_, *p* = 4 × 10^−9^), to a greater degree than motif-only or background genes (Figure S7D). Thus, across an independent cohort of roughly 10,000 human tumors, the RNA targets engaged by mut p53 in our system are elevated in TP53-mutant cancers, supporting a 3′UTR-level regulatory role for mut p53 that generalizes across the broad class of DNA-binding-domain mutants.

## DISCUSSION

Mut p53 is a common feature across cancers with a strong genetic selection for a small number of somatic mutants in the DNA-binding-domain. The role of these mutations is a topic of much debate in the literature, with strong recent evidence being presented that in mature cancer cell lines containing only the mutant no phenotypic difference was observed when the mutant protein was knocked out using CRISPR-Cas9.^61^ Despite this, the strong selective pressure for a small subset of mutants suggests they likely play a role in another aspect of carcinogenesis, such as oncogenic transformation or loss of heterozygosity.

In this study, we describe a biochemical analysis of the varied interactions exhibited by both WT and mut p53, and how DNA-binding-domain mutations redirect p53 localization and function. Our data clearly show that these mutations promote certain situational functions of p53, such as modulating mitochondrial stress and RNA processing. While these functions are part of the complex repertoire of WT p53 phenotypes, they appear to be overrepresented when the protein can no longer bind to DNA. We posit that once the strong DNA-binding affinity of WT p53 is lost or diminished, the balance of p53 activity shifts toward cytoplasmic, RNA-associated functions, promoting its translocation and association with structured RNA and RNA-binding proteins. Whether the mutations also confer qualitatively altered RNA-binding properties remains to be determined, as direct biochemical comparison of WT and mut p53 RNA-binding affinity was not performed in this study.

Preliminary experiments indicate that truncation of the C-terminal domain—previously shown to mediate sequence-nonspecific nucleic acid binding and to be required for RNA binding by p53^62,63^—shifts both WT and mut p53 toward nuclear localization, with a concomitant loss of *ds*-RNA- and ZC3H7B-proximity signal by PLA (Figure S8), though whether this reflects reduced RNA/protein-binding capacity or simply altered subcellular access due to the truncation could not be distinguished and remains an open question for future work.

While it remains unclear how these particular interactions could promote oncogenic transformation at the early stages of cancer, our datasets provide a biochemical basis for such investigations and provide commonalities between different hotspot mutations.

### Limitations of the study

Despite these insights, our study contains several limitations. First, the inteinbased method we employ in this study requires the use of permeabilized cells, which disrupts the cellular environment and limits its ability to capture cytosolic interactions, leaving a gap in our knowledge. Second, we perform these experiments in a non-cancer-derived model cell line, HEK293T, which functionally suppresses endogenous p53. While this allows for the expression of all variants of p53 in a single controlled system, it does not capture the unique environment that each mutation is typically found in. However, preliminary exploration of key observations (mitochondrial function, monosome profiling, and Seahorse assay) in a second model (HCT116 p53^−/−^) suggests that our hypotheses are not limited to HEK293T cells (Figure S9). Future work will focus on exploring mut p53 as an RBP in representative cancer models.

## RESOURCE AVAILABILITY

### Lead contact

Requests for further information and resources should be directed to and will be fulfilled by the lead contact, Ciaran P. Seath (cseath@cornell.edu).

### Materials availability

All unique/stable reagents generated in this study are available from the lead contact with a completed materials transfer agreement.

### Data and code availability

- Mass spectrometry data have been deposited at MassIVE: MSV000099926 and are publicly available as of the date of publication.
- Sequencing data have been deposited at NCBI Gene Expression Omnibus: CLIP-seq (GEO: GSE310613), RNA-seq (GEO: GSE310614), miRNA-seq (GEO: GSE310615), and Ribo-seq (GEO: GSE335668) and are publicly available as of the date of publication.
- This paper does not report original code.
- Any additional information required to reanalyze the data reported in this paper is available from the lead contact upon request.

## STAR★METHODS

### EXPERIMENTAL MODEL AND STUDY PARTICIPANT DETAILS

HEK 293T cells and stable HEK293T cells expressing mutant and wt p53 were cultured as a monolayer in DMEM, supplemented with 10% v/v FBS, 100 U mL^−1^ penicillin, and 100 μg mL^−1^ streptomycin. Cells were maintained in an incubator at 37°C with 5% CO2. For stable cell lines, media was supplied with 1.5 μg/mL Puromycin (InvivoGen, ant-pr-1). HEK293T cells were provided as a gift from the MacMillan laboratory (Princeton University) and were derived from a female human embryonic donor (Cellosaurus CVCL_0063); this stock was not independently authenticated in this study.

HCT116 and HCT116 p53 KO cells were cultured as a monolayer in McCoy’s 5A medium (ThermoFisher, 16600082), supplemented with 10% v/v FBS, 100 U mL^−1^ penicillin, and 100 μg mL^−1^ streptomycin. HCT116 cells are derived from a 48-year-old male colon carcinoma patient (Cellosaurus CVCL_0291). HCT116 p53 KO cells (Abcam, ab289184) were validated by the manufacturer via next-generation sequencing, with a matched wild-type control line provided.

All cell lines were tested monthly for mycoplasma contamination using the MycoStrip Mycoplasma Detection Kit (InvivoGen, repmysnc-50), an isothermal PCR-based lateral-flow assay, and all results were negative. As this study used established, immortalized human cell lines rather than primary human tissue or animal models, no additional institutional (IRB/IACUC) oversight was required; sex-specific effects were not assessed, as both lines are derived from single donors of fixed sex and findings should not be generalized across sexes without further validation.

### METHOD DETAILS

#### Cloning

To construct p53 plasmid conjugated to intein tags, pcDNA3.1(+)-H3.1-Cfa^N^ plasmid (generous gift from Dr. D.W.C. MacMillan) was PCR cloned for backbone and intein expressing CDS using Phusion polymerase (New England Biolab (NEB), M0536S). The backbones were then fused with PCR amplified CDS region from commercial plasmid expressing wt p53 and ligated using In-Fusion snap assembly kit (Takara Bio, 638947). The encoded constructs were thus under the control of a CMV promoter. Ligated vectors were introduced to NEB 5-alpha competent E. coli cells (NEB, C2987H) by heat shock transformation. For mutant p53 expressing plasmids, mutagenesis was performed individually for every mutant using Q5 Site-Directed Mutagenesis Kit (NEB, E0554). All primers were designed using SnapGene (version 7.2) and purchased from Integrated DNA Technologies (IDT). Ligated pcDNA3.1(+) vectors were introduced to NEB 5-alpha competent E. coli cells (NEB, C2987H) by heat shock transformation. The whole plasmid sequencing was performed by Plasmidsaurus using Oxford Nanopore Technology with custom analysis and annotation. For a detailed list of plasmids used in this study, refer to Table S3.

#### Transient transfection

Each 10 cm plate of HEK293T cells at 50% confluency was transfected with a plasmid encoding mutant or wt p53 (5 μg per plate) with 15 μL of Lipofectamine 2000 (Thermo, 11668019) following the manufacturer’s instructions. After 6 h, the media was aspirated and replaced with fresh media. Transfection was performed for 24 h in an incubator at 37°C with 5% CO_2_.

#### Generation of stable cell line

Concentrated lentivirus (>10^8^ TU/mL) that expresses wt and mutant p53 was prepared by VectorBuilder. Briefly, to construct stable cell line, low passage HEK293T cells (24-well plates, 50% confluency) were incubated with optimal amount of lentivirus particles to achieve a MOI = 4 for 24 h. Incubating media were supplied with 5 μg/mL of Polybrene (Millipore Sigma (Sigma), TR-1003-G). Virus-containing medium was replaced with complete DMEM medium after 24 h and cells were incubated for another 24 h. Selective medium containing 1.5 μg/mL Puromycin (InvivoGen, ant-pr-1) were then replaced, and cells were maintained in the selective medium for 4 days to allow for complete selection.

#### RNA-seq analysis

Stable cells that express wt and mutant p53 from three different biological replicates were harvested, pelleted, and lysed with DNA/RNA Shield (Zymo Research, 1100–50) (5 × 10^5^ cells/50 μL). Lysed cells were submitted to Plasmidsaurus for library construction and RNA-seq analysis. In brief, FastQ was generated and demultiplexed with BCL Convert v4.3.6 and fqtk v0.3.1. Read-filtering was performed using FastP v0.24.0 with the following criteria: poly-X tail trimming, 3′ quality-based tail trimming, a minimum Phred quality score of 15, and a minimum length requirement of 50 bp. Alignment to the appropriate reference genome was done using STAR aligner v2.7.11 with non-canonical splice junction removal and output of unmapped reads. Coordinate sorting of BAM files was performed using samtools v1.22.1. Removal of PCR and optical duplicates was performed using UMICollapse v1.1.0. Alignment quality metrics, strand specificity, and read distribution across genomic features were performed using RSeQC v5.0.4 and Qualimap v2.3. Comprehensive QC report was generated using MultiQC v1.32. Gene-expression quantification was performed using featureCounts (subread package v2.1.1) with strand-specific counting, multi-mapping read fractional assignment, exons and three prime UTR as the feature identifiers, and grouped by gene_id. Final gene counts were annotated with gene biotype and other metadata extracted from the reference GTF file. Sample-sample correlations for sample-sample heatmap and PCA were calculated on normalized counts (TMM, trimmed mean of M-values) using Pearson correlation. Differential expression using edgeR v4.0.16 with filtering for low-expressed genes with edgeRfilterByExpr with default values. Functional enrichment performed for human and mouse samples using gene set enrichment analysis with GSEApy v0.12 using the MSigDB Hallmark gene set. Results were visualized with GraphPad Prism, and cross-analyzed with other omics datasets with R studio. Refer to Table S4 for the full dataset.

#### Label free global proteomics

Stable cells expressing mutant or wt p53 from three independent biological replicates were lysed with SDS lysis buffer, and proteins were cleaned up, and digested using Sera-Mag Carboxylate SpeedBeads (Cytiva Life Sciences: E7 Cat. 45152105050250 and E3 Cat. 65152105050250) following manufacturer’s protocol. Digested peptides were split into two technical replicates per sample.

Peptide digests were acidified with TFA to 0.1% (v/v) and desalted using 2 μg capacity ZipTips (Millipore, Billerica, MA) according to manufacturer instructions. Following drying under vacuum, peptides were re-solubilized in 0.1% formic acid (FA) to a final concentration of 100 ng/μL. Samples were analyzed on a nanoElute 2 (plug-in V2.1.79.0 (1); Bruker, Bremen, Germany) coupled to a Bruker TimsTOF Pro 2 mass spectrometer (Breman, Germany), equipped with a CaptiveSpray source and a 20μm zero dead volume (ZDV) Sprayer. Peptides (corresponding to 100 ng) were loaded onto a Thermo Fisher (Waltham, MA) PepMap *Neo* C18 trap column (300 mm × 5 mm, 5mm particle size) and then separated on an Aurora Series Elite reverse-phase C18 column (15 cm × 150 μm, 1.7μm particle size) from IonOpticks (Fitzroy, Australia). The column temperature was maintained at 50°C using an integrated Bruker Column Toaster (Bremen, Germany). The column was equilibrated using 4 column volumes at 800 bar before loading samples in 100% buffer A (99.9% Fisher Optima LC/MS water, 0.1% FA) at 201.6 bar. The trap column was equilibrated at 201.6 bar. Samples were separated at 500nL/min using a linear gradient from 2% to 35% buffer B (99.9% Fisher Optima LC/MS acetonitrile, 0.1% FA) over 20.0 min before ramping to 95% buffer B (in 0.5 min) and sustained at 95% buffer B for 4.5 min (total separation method time 25.0 min). The Bruker TimsTOF Pro 2 was operated in DIA-PASEF mode using Tims Control v. 5.0.2. Settings for the MS method were as follows: Mass Range 100 to 1700m/z, 1/K0 Start 0.6 V. · /cm2 End 1.4 V · s/cm2, TIMS Ramp and accumulation time 75ms, Capillary Voltage 1700V, Dry Gas 3 L/min, Dry Temp 200°C, DIA-PASEF settings: 18 MS/MS scans (50m/z windows, 0.21 1/K0 windows, total cycle time 0.74), mass range 300–1200, and CID collision energy 20eV (at 0.60, 1/K0) to 65eV (at 1.60, 1/K0). The analysis was performed at The Herbert Wertheim UF Scripps Institute for Biomedical Innovation & Technology, Mass Spectrometry and Proteomics Core Facility (RRID:SCR_023576).

Data was processed via DIANN 1.8.1. Parameters set as follows: trypsin/P digestion, 3 missed cleavages, 3 max. variable modifications, N-term M excision, Ox(M), Ac(N-term) and C carbamidomethylation. Peptide length range was 7–30, precursor charge range 1–4, m/z range 300–1800, and fragment ion range 200–1800. Mass accuracy and MS accuracy were both set to 10. The following settings on the algorithm were checked: “Use isotopologues”, “MBR”, “No shared spectra”, “Heuristic protein inference”. Precursor FDR was set to 1%. A spectral library was generated via DIANN from all known human proteins (In-Silico spectral library). Resulting matrix.pg file was opened in Perseus (v2.0.7.0). Intensities were inputted as “main”, the rest of the descriptors are categorical. Data was then transformed (Log_2_). Data is annotated by treatment. Missing values were imputed with Perseus default settings. Normalization was performed via median subtraction. Following this process, a volcano plot was generated utilizing a *t* test for statistical significance. The resulting volcano plots were plotted in GraphPad Prism 10 for final figures. Metascape and STRING was used for Gene Ontology. Refer to Tables S1 and S2 for the full dataset.

#### General procedure for photoproximity labeling in cells

Peptides were prepared and purified as previously described.^17^ 3×10^7^ HEK 293T cells transfected with p53-HA-Cfa^N^-FLAG were permeabilized by hypotonic pressure with 3 mL RSB buffer (10 mM tris, 15 mM NaCl, 1.5 mM MgCl_2_, 1 × Halt protease cocktail, pH 7.6) for 10 min on ice. The cells were pelleted by centrifugation at 400*g* for 5 min at 4°C. The cells were resus-pended in 3 mL RSB buffer, and homogenized with ten strokes of a loose pestle Dounce homogenizer, and pelleted at 400 g for 5 min at 4°C. The cells were resuspended in crosslinking buffer (20 mM HEPES, 1.5 mM MgCl_2_, 150 mM KCl, 1 × Halt protease cocktail, pH 7.6) and centrifuged at 400 g for 5 min at 4°C. Finally, the cells were resuspended in 300 mL of crosslinking buffer per 1 × 10^7^ cells. To the suspended pellet was added Cfa^C^-Ir in crosslinking buffer (0.5 μM final concentration). For splicing efficiency confirmation, Cfa^C^-Bt was added in crosslinking buffer (0.25 μM final concentration). The cell suspension was incubated at 37°C for 1 h.

The cells were isolated by centrifugation at 400*g* for 5 min at 4°C and washed twice with crosslinking buffer (500 mL) to remove excess peptide. The pellets were then resuspended in 3 mL crosslinking buffer containing diazirine-biotin conjugate (200 μM) and irradiated with blue light for 60s in the Penn PhD Photoreactor M2 at 100% light intensity at 4°C. The cells were re-isolated by centrifugation at 400*g* for 5 min at 4°C and washed once with crosslinking buffer to remove excess biotin-diazirine. For specificity test, wells at this step were cytospun onto poly-L-lysine-coated glass coverslips at 400 g for 5 min, then fixed, permeabilized, and stained as described in the immunofluorescence assay of p53-expressing cells section. The washed pellets were then resuspended in 2 mL LB3 buffer (10 mM tris, 100 mM NaCl, 1 mM EDTA, 0.5 mM EGTA, 0.1% sodium deoxycholate, 0.5% sodium lauroyl sarcosinate, pH 7.5) and sonicated using a Diagenode Bioruptor Plus for 12 cycles of 30 s on and 30 s off at high power at 4°C. For splicing confirmation, pellets were collected and lysed as described immediately after the intein washing step and then subjected to western blot analysis.

The lysed cells were then clarified through centrifugation at 15,000 g for 20 min at 4°C and the protein concentration of the super-natant was determined by Pierce BCA assay. Protein concentration was normalized across all experimental replicates and diluted to 1 mg/mL with binding buffer (25 mM tris, 150 mM NaCl, 0.25% v/v NP-40, pH 7.5). 1 mL of each sample was then incubated with 100 μL of pre-washed magnetic Sepharose streptavidin beads for 2 h at RT with end-over-end rotation. The beads were subsequently washed twice with 1% w/v SDS in PBS, twice with 1 M NaCl in PBS, and 10% EtOH in PBS × 3. At this point proteins can be eluted from beads for western blotting by addition of 1 × Laemmli sample buffer (Biorad, 1610747) containing 20 mM DTT and 25 mM biotin and heated to 95°C for 10 min.

#### Western blot analysis

Cell lysates were quantified with BCA and normalized to the same concentration and the cell lysate was boiled in 1X Laemmli buffer (diluted from 4x Laemmli Sample Buffer, supplied with 2.5% v/v β-mercaptoethanol) at 95°C for 10 min and loaded to a pre-cast Bolt 4–12% Bis-Tris Plus Mini Protein Gel (Thermo, NW04120BOX). Proteins were transferred to a nitrocellulose membrane (Thermo, 88018) and blocked with 2% Bovine Serum Albumin (BSA) (Fisher Scientific (Fisher), BP9704100) in TBST (diluted from 10x TBST (Bioland Scientific LLC, TBST01–03) with distilled water) for 1 h at RT. Membranes were then stained with primary antibodies diluted in 2% BSA in TBST at 4°C overnight on a rocker. Membranes were then washed 3 × 10 min with TBST and stained with secondary fluorescent antibody diluted in 2% BSA in TBST for 1 h at RT. Membranes were then washed with TBST for 3 × 10 min and then imaged on Licor Odyssey CLx scanner. Quantification of the membrane was performed with Fiji (v1).

#### Label-free proteomics and data analysis of enriched sample

Following streptavidin-enrichment, the beads were resuspended in 0.5 mL PBS and transferred to a new tube. The beads were washed with 3 × 0.5 mL PBS and 3 × 0.5 mL 100 mM ammonium bicarbonate (Sigma, A6141). The beads were resuspended in 0.5 mL of 3 M urea (Sigma, U5378) in DPBS and 25 μL of 200 mM DTT in 25 mM ammonium bicarbonate was added. The beads were incubated at 55°C for 30 min. Subsequently, 30 μL of 500 mM iodoacetamide in 25 mM ammonium bicarbonate was added and incubated for 30 min at room temperature in the dark. The supernatant was removed and the beads washed with 3x with 0.5 mL of DPBS and 6x with 0.5 mL of 50 mM ammonium bicarbonate. The beads were resuspended in 0.5 mL of 50 mM ammonium bicarbonate and transferred to a new tube. The beads were resuspended in 40 μL of 50 mM ammonium bicarbonate added with 1.2 μL of trypsin (1 mg/mL in 50 mM acetic acid (Fisher, A11350)), and incubated overnight with end-over-end rotation at 37°C. After 16 hr, a further 0.8 μL of trypsin was added and the beads were incubated for an extra 1 h at 37°C. Supernatant was then transferred to new tube and each biological replicate split into two technical replicates. Three biological replicates were used per condition. Peptides were treated and analyzed as described above.

For data analysis across different mutants, significant hits were determined by FDR<0.05. The GO term enrichment analysis was performed using Metascape (metascape.org) on “gained” interactions and “lost” interactions protein hit lists derived from proximity labeling experiments for each of the five hotspot p53 mutants, with a cutoff of LFC>0.5. Heatmaps displaying the top enriched GO biological process terms and their −log_10_(*p*-values) across mutants were generated directly by Metascape using the multiple gene list analysis mode. For each mutant, enriched GO biological process terms were extracted along with their −log_10_(*p*-values). The resulting enrichment matrices (GO terms × mutants) were compiled across all mutants. Principal component analysis (PCA) was performed in R using the prcomp function with scaling, on the transposed matrix (mutants as observations, GO terms as features), to visualize the similarity of “gained” and “lost” interaction profiles across hotspot mutants.

#### Procedure for immunoprecipitation MS

3×10^7^ HEK 293T cells transfected with wt/mutant p53-HA-Cfa^N^-FLAG grown in 15cm plates were washed 2–3x with ice-cold PBS, then lysed directly in the plate with EBC buffer (50mM Tris-HCl, 120mM NaCl, 0.1mM EDTA, 0.5% NP-40, 10% glycerol, 1× Halt protease inhibitor cocktail) and harvested via scraping. After lysing for 30 min on ice, the lysates were sonicated on the Diagenode Bioruptor Sonicator (LOW, 5 cycles at 4°C, 7 s ON, 5 s OFF). Samples were then centrifuged (12,000 × g, 15 min, 4°C), the supernatant was harvested, and all lysates were normalized to 1 mg/mL. 5 μg of the anti-FLAG antibody and isotype control antibody were then conjugated to Pierce Protein A Magnetic Beads (Thermo Scientific, 88845) according to the manufacturer’s instructions. The lysates were then incubated with the prepared protein A beads overnight at 4°C with rotation. Beads were washed 4x with Wash buffer (50mM Tris-HCl, 150mM NaCl, 5mM EDTA, 0.1% Tween 20) and transferred to new tubes. Proteins were eluted with 1% SDS in RIPA buffer at 95°C for 10 min. Eluted protein was then precipitated on to speedbeads, washed and digested as described in the Label-free proteomics protocol above.

#### Procedure for cell fractionation

One 10 cm plate of HEK 293T cells was transfected with the p53 plasmids and cells were collected with trypsin. Cells were lysed and fractionated into cytosolic, membrane, and nuclear fractions using the Subcellular Protein Fractionation Kit for Cultured Cells (Thermo Fisher Scientific, 78840) according to the manufacturer’s instructions. Fraction identity and purity were confirmed by western blotting to GAPDH, EGFR, and c-Jun.

#### Immunofluorescence assay of p53-expressing cells

Stable cells or transiently transfected HEK293T cells that express p53 protein were plated on pre-treated round coverslips with poly-L-lysine (Sigma, P4707) in 24-well plates. When the cells reached 80% confluency (24 h post-transfection) they were gently washed with DPBS, then immediately fixed with 4% paraformaldehyde in PBS (Thermo, AAJ61899AP) for 10 min at RT and blocked and permeabilized for 1 h at RT with 2% BSA in PBST solution (1X DPBS, added with 0.1% Triton X-100 (Fisher, 501781842)). Cells were then stained with primary antibody diluted in 2% BSA in PBST solution at 4°C ON. Following primary antibody staining, cells were washed with DPBS three times and stained with secondary fluorescent antibody for 1 h at RT. Coverslips were mounted with mounting media supplied with DAPI (Fisher, NC9524612) and sealed with clear nail polish on glass slides. Slides were imaged on an Olympus FV3000 laser scanning microscope with Abbelight system with a 100X UPLAPOHR OIL NA 1.5 WD 0.12 mm lens, with 405, 488, 561, and 640 nm laser. Colocalization signal analysis and line analysis was done via Fiji (ver 1) and CellProfiler (version 4.2.8).

#### PLA analysis

Transiently transfected HEK293T cells that express p53 protein (for ZC3H7B, dsRNA, and COX7C), or stable cells that expresses p53 constructs (for TOMM70 and AKAP1 interaction) were plated on pre-treated round coverslips with poly-L-lysine in 24-well plates and fixed with 4% PFA in PBS 24 h post-transfection. Cells were incubated with pre-chilled 100% MeOH at −20°C to permeabilize. Once fixed and permeabilized proximity ligation was carried out as recommended by manufacturer using the Duolink *in situ* red starter kit mouse/rabbit (Sigma-Aldrich DUO92101). For transient transfected cells, slides were stained with primary HA-tag antibody ON at 4°C to identify p53-expressing cells, followed by 1 h of secondary antibody staining at RT. Slides were then mounted with duolink *in situ* mounting media with DAPI and sealed with clear nail polish for imaging. Slides were imaged on an FV3000 laser microscope as being described previously. At least 3 images were taken of each slide in different position. Numbers of PLA puncta signal were quantified using CellProfiler nuclear speckle counting software. Statistical significance was determined by a one-way ANOVA test followed by post hoc Dunnett’s multiple comparisons test of foci per cell using GraphPad Prism.

#### miRNA-seq of p53-expressing cells

HEK cells were seeded and transfected in 10 cm plates as described above. The next day, cells were harvested with trypsin, washed with PBS, and RNAs were extracted from 5 × 10^6^ cells using the RNeasy plus mini kit (Qiagen, 74134) according to the manufacturer’s instructions. Total RNA was submitted to The Herbert Wertheim UF Scripps Institute for Biomedical Innovation & Technology - Genomics Core (RRID:SCR_017827), where it was quantified using a Qubit 2.0 Fluorometer (Invitrogen, Carlsbad, CA) and evaluated on an Agilent 4200 TapeStation (Agilent Technologies, Santa Clara, S10 CA) for quality assessment. All RNA samples had RNA Integrity Number (RIN) ≥7.0 and were used for small RNA library preparation. RNase-free working environment was maintained, and RNase-free tips, tubes, and plates were utilized. 0.4 μg of total RNA per sample was used. The library preparation from the input RNA was conducted according to the NEXTFLEX small RNA kit v4 (Revvity, NOVA-5132–31). Briefly, the input RNA has been ligated to 3′ adenylated adapter and 5′ adapter. The RNA was then reverse transcribed to generate the first strand of cDNA. The synthesized product was cleaned up with beads and amplified with PCR (using 15 cycles) to incorporate a unique barcode and to generate final libraries. The libraries were purified using SPRI beads to remove any remaining primers and adaptors. The final libraries were validated on an Agilent 4200 TapeStation (Agilent Technologies, Santa Clara, CA), normalized to 4 nM, pooled equally, and loaded onto an Illumina NextSeq 2000 using P2 Reagents (100 Cycles) v3. (Cat. No: 20046811) kit at 700pM final concentration with 1% PhiX and sequenced using 2 × 51bp paired-end chemistry, with 8 bp index. On average, a 20.6 million reads pass filter were generated per sample. miRNA sequencing data were processed using the nf-core/smrnaseq pipeline^64^ (version 2.4.0, doi: https://doi.org/10.1038/s41587-020-0439-x.) on the HiPerGator high-performance computing cluster with Nextflow (version 25.04.4) and Singularity (version 3.10.4). Raw FASTQ files were quality-checked with FastQC (version 0.12.1) and trimmed using cutadapt^65^ (version 5.2) following the instruction of NEXTFLEX small RNA kit v4. Contamination was filtered with Bowtie2 (version 2.5.4). Reads were aligned to the miRbase mature miRNA and miRbase hairpin using Bowtie1 (version 1.3.1). Post-alignment processing of miRbase hairpin was done with SAMtools (version 1.2.1), edgeR (version 4.8.0), and mirtop (version 0.4.28). Alignment against human reference genome was performed with Bowtie1. MiRDeep2 (version 0.1.3) was used to perform novel and known miRNA discovery. Mirtrace (version 1.0.1) was adapted for miRNA quality control, and MultiQC (version 1.27.1) was used for raw read, alignment, and expression results presentation. Downstream analysis was performed with DESeq2 (v1.34.0) to identify differentially expressed (DE) genes. Low count miRNA were filtered out (total sum count in all replicates less than 10). Statistical significance was determined with pAdjusted <0.1 for both miRNA and hairpin. Data was visualized using R studio. Refer to Table S5 for the full dataset.

#### Crosslinking immunoprecipitation (CLIP) and CLIP-seq analysis

Cells were washed twice with 1× PBS and UV-crosslinked on ice with one pulse of 400 mJ/cm^2^ followed by one pulse of 200 mJ/cm^2^. Cells were immediately scraped in fresh ice-cold 1× PBS and centrifuged at 5300 × *g* for 5 min at 4°C. The resulting pellets were flash-frozen and stored at −80°C. Two biological replicates were prepared, each from a single 15-cm dish. Cell pellets were resus-pended in 1 mL of lysis buffer (1× PBS, 0.1% SDS, 0.5% sodium deoxycholate, 0.5% NP-40, supplemented with freshly added protease inhibitors), and immunoprecipitations were performed 2 h at 4°C using a mouse monoclonal anti-FLAG antibody (8 μg conjugated to 100 μL of Protein G Dynabeads (Invitrogen, Cat # 10003D)). Subsequent radiolabeling, RNA extraction, reverse transcription, and qPCR steps were performed as described previously^66^ with minor modifications. During the protein digestion step, a 7 M urea incubation was introduced prior to RNA precipitation, as described by Ule et al.^67^ Wild-type and mutant samples were pooled after reverse transcription barcoding to increase yield and ensure uniform amplification. Libraries were sequenced on an Illumina NextSeq platform to generate 75-nt single-end reads.

Sequencing reads were processed using the CLIP Tool Kit (CTK)^68^ following published guidelines.^69^ Briefly, reads were quality-filtered, demultiplexed, trimmed to remove 5′ and 3′ linker sequences, and collapsed to eliminate PCR duplicates. Filtered reads were then aligned to the human genome (hg38) using BWA.^70^ Mapped reads were further collapsed using fastq2collapse.pl from the CTK toolkit with default parameters. The genomic distribution of uniquely mapped CLIP reads was determined using bed2annotation.pl. Uniquely mapped CLIP tags from all samples were pooled, and peaks were identified using tag2peak.pl (CTK) with a significance cutoff of *p* < 0.05. For each sample, tag counts within significant peaks were quantified using the countOverlaps function in R. Differentially bound peaks between mutant and wild-type p53 were identified using DESeq, applying an adjusted *p*-value threshold of <0.05. Refer to Table S6 for the full dataset.

#### RT-qPCR assay

For each independent biological replicate, 2 × 10^6^ cells were harvested with trypsin, washed with PBS, and RNAs were extracted using RNeasy plus mini. The RNA samples were treated with DNase (Invitrogen, AM1907) to remove genomic DNA. The RNA concentrations were measured using Qubit RNA BR Assay Kit (Thermo Scientific, Q10210) and normalized to the same concentration. cDNAs were synthesized with the SuperScript III First-Strand Synthesis System (Invitrogen, 18080051) according to the manufacturer’s protocol with random hexamers. The cDNA concentration was measured with Qubit ssDNA Assay Kit (Thermo Scientific, Q10212) and normalized to 5 ng/μL. 10 ng of cDNA was loaded in real-time qPCR reactions, which were performed in technical duplicates using the Power SYBR Green PCR Master Mix (Thermo Scientific, 4367659) and specified primer (*COX7C*: forward CTTCCAGCAGCGGTATGTT reverse-AGCTAGTAACGACCACTTGTTT; *GAPDH*: forward- AATCCCATCACCATCTTCCAG reverse-CCTTCTCCATGGTGGTGAAGAC). Quantification of gene expression was measured using the 5 Real-Time PCR System. Statistical analysis and data visualization was performed with GraphPad Prism.

#### Seahorse assay

Mitochondrial respiration analysis (Seahorse assay) was performed at the Metabolic core at The Herbert Wertheim UF Scripps Institute for Biomedical Innovation. Per individual well in XF Pro Cell Culture Microplates (Seahorse XFe96/XF Pro FluxPak, Agilent Technologies, 103792–100) pre-treated with poly-L-lysine, 2 × 10^4^ stable HEK293T cells expressing p53 constructs, or 2.5 × 10^4^ HCT116 p53^−/−^cells transiently expressing p53 constructs 32 h post-transfection, were plated 16 h prior to assay performing. To assess mitochondrial respiratory function, in whole cells, the growth media was replaced with XF media (unbuffered DMEM, pH 7.4, Agilent Technologies, 103680–100) with 10 mM pyruvate and 2.5 mM glucose supplied. The cell plate was equilibrated for 30 min at 37°C in a CO_2_-free incubator before being loaded in the XF-96 analyzer. Inside the analyzer, the plate was combined with the pre-calibrated XF cartridge with the individual well ports A, B and C loaded with 10× concentrated stocks of the following compounds, respectively: A. Oligomycin (20 μg/mL); B. FCCP (30 μM – HEK stable cells, 5 μM – HCT116 cells. Concentration was determined in preliminary experiments with titration); C. Rotenone and Antimycin A (20 μM each). Oxygen consumption rate (OCR) was monitored in real-time at 37°C under basal conditions and following the sequential delivery of the compounds in the cartridge ports. Non-mitochondrial rote-none/antimycin-insensitive OCR was subtracted from all other OCR measurements. Hoechst staining was performed post-assay at 5 μg/mL for 15 min, and images of each well were acquired using the ImageXpress HCS.ai high-content screening system with MetaXpress Acquire Software 2025.1. Cell counts per well were analyzed using InCarta (v2.8) and used for normalization in Wave software (v2.6.4.39).

#### Mitotracker labeling

Per individual well in 12-well glass bottom plate (0.17 mm) (Cellvis, P12–1.5H-N), 3 × 10^5^ stable cells expressing p53 constructs were plated 16 h prior to assay performing. Cells were treated with warm media supplied with MitoTracker at 37°C for 30 min. A final concentration of 250 nM MitoTracker Red (Thermo, M22426), 100 nM MitoTracker Green (Thermo, M7514), or 100 nM MitoTracker Orange (Thermo, M7511) were used. Hoechst dye (Thermo, H3570, 1 μg/mL) was added during the final 10 min of incubation time. Cells were immediately imaged on an Olympus FV3000 laser scanning microscope with Abbelight system with a 100X UPLAPOHR OIL NA 1.5 WD 0.12 mm lens, with 405, 488, 561, and 640 nm laser. At least 3 images were taken from two independent biological replicates. Signal analysis was done via Fiji. Images were transformed to 8-bit, thresholding was performed with Default ignore black white method, and intensity was measured for quantification. Statistical significance was determined by a one-way ANOVA test followed by post hoc Dunnett’s multiple comparisons test of foci per cell using GraphPad Prism.

#### Analysis of TCGA tumors

##### Data sources

*TP53* somatic mutation calls were obtained from cBioPortal for the TCGA PanCanAtlas studies (32 studies; protein-level HGVSp annotations). Gene-level RNA-seq (batch-corrected, log_2_) and mature-miRNA expression matrices were obtained from the UCSC Xena PanCanAtlas hub; total-p53 protein from the TCGA reverse-phase protein array (RPPA) dataset; and ABSOLUTE tumor-purity estimates from the NCI Genomic Data Commons. Solid-tissue-normal samples were excluded, leaving 10,323 tumors across 33 cancer types.

##### Stratification

Each tumor was assigned to a *TP53* stratum by its most severe allele: wild-type, DNA-binding-domain (DBD) missense (residues 94–312), non-DBD missense, or truncating/null (nonsense, frameshift, or splice-site).

##### Differential expression by *TP53* status

For each mature miRNA and each mRNA, expression was modeled by ordinary least squares as value ~ C(*TP53* status, reference = wild-type) + C(cancer type), with cancer type included to control for tissue of origin. The reported coefficient (β) is the mean shift relative to wild-type, with Benjamini–Hochberg-adjusted *q* values. Group medians were additionally compared by Kruskal–Wallis with Dunn post-hoc tests.

##### p53 protein dependence

Within *TP53*-mutant tumors, mature miR-139–5p was modeled against total-p53 protein (RPPA) with cancer type and ABSOLUTE purity as covariates. Mutant-specificity was assessed across all tumors by an RPPA-p53 × mutant-status interaction term in the same covariate framework. For display (Figure S7), tumors were binned into quintiles of p53 protein within wild-type and mutant groups, and the median miR-139–5p (±s.e.m.) plotted per bin.

##### RNA consensus motif

The mutant-p53 RNA consensus (the GGCUGG-centered motif identified by CLIP) was searched, on both strands, in human 3′UTR sequences (TargetScan), miRNA sequences (miRBase v22), and pri-miRNA flanking regions (Ensembl). A gene was scored motif-positive if its 3′UTR contained ≥1 occurrence. Enrichment of the motif in mutant-p53-bound versus non-bound transcripts was tested by Fisher’s exact test, and the association between motif copy number and tumor expression was tested while controlling for 3′UTR length and GC content.

##### CLIP-target integration

Mutant-p53-bound 3′UTR transcripts were defined from the differential CLIP-seq peak tables as genes carrying a 3′UTR peak with greater mean signal in the mutant than in wild-type cells. For each gene, a tissue-adjusted expression difference (DBD-missense – wild-type) was computed as the cancer-type-weighted mean of within-cancer-type group differences. The distributions of this difference for background, motif-positive, CLIP 3′UTR-bound, and CLIP-and-motif-positive gene sets were compared by Mann–Whitney *U* test against background.

##### Software

Analyses were performed in Python (pandas, numpy, scipy, statsmodels); gene sets used MSigDB Hallmark and MitoCarta3.0 inventories. Figures were prepared in GraphPad Prism and matplotlib. All input datasets are publicly available.

#### Ribosome profiling

HEK293 cells expressing the wt, G245S, or R273H p53 were grown to 80% confluency in 10 cm dishes. Three biological replicates were prepared for each cell line from independent 10 cm dishes. Cells were treated with cycloheximide (Millipore Sigma, C1988) for 2 min at 37°C and 5% CO_2_, then rapidly washed and harvested. Washes were performed with ice-cold polysome gradient buffer containing 20 mM Tris, pH 7.5, 150 mM NaCl, 5 mM MgCl_2_, freshly supplemented with 1 mM DTT and 100 μg/mL cycloheximide. Cells were lysed directly on plates on ice in 500 μL ice-cold polysome lysis buffer containing 20 mM Tris, pH 7.5, 150 mM NaCl, 5 mM MgCl_2_, 1% Triton X-100 (Thermo Fisher, 85111), freshly supplemented with 20 U/mL SUPERase-In RNase inhibitor (Thermo Fisher, AM2694), 24 U/mL TURBO DNase (Thermo Fisher, AM2239), 1 mM DTT, 100 μg/mL cycloheximide, and 1× EDTA-free pro-tease inhibitor cocktail (Millipore Sigma, 11836170001). Lysates were clarified by centrifugation at 20,000 × g for 2 min at 4°C and were either flash-frozen or used immediately for monosome fractionation.

For monosome fractionation, lysates were supplemented with 5 mM CaCl_2_ and digested with micrococcal nuclease at 3 U/μg RNA for 30 min at room temperature. Digestion was quenched by addition of EGTA to a final concentration of 6.25 mM, and samples were loaded onto sucrose gradients as previously described.^71^ Gradients were centrifuged for 2 h at 41,000 rpm in an SW-41 rotor (Beckman Coulter), and monosome fractions were collected using a BioComp gradient fractionation system coupled to a Gilson FC203B fraction collector and Triax software (BioComp Instruments).

RNA was extracted from collected monosome fractions using TRIzol reagent according to the manufacturer’s instructions. Ribo-some-protected fragment libraries were prepared using barcoded linkers as previously described.^72^ Indexed libraries were pooled and sequenced on an Illumina NextSeq 2000 Sequencing System using a P3 flow cell.

#### Ribo-seq analysis

FASTQ file integrity and formatting were assessed using FQ lint, and initial read quality was evaluated using FastQC. Adapter trimming and quality filtering were performed using Trim Galore, which wraps Cutadapt and FastQC. Where applicable, unique molecular identifiers were extracted using UMI-tools, and ribosomal RNA reads were removed using SortMeRNA. Processed reads were aligned to the human GRCh38 reference genome and transcriptome using STAR, and resulting alignment files were sorted, indexed, and summarized using SAMtools. Ribo-seq-specific quality-control metrics, including read length distribution, metagene profiles, P-site offset estimation, and trinucleotide periodicity, were generated using Ribo-TISH^73^ and Ribotricer.^74^ Library quality was further confirmed by strong trinucleotide periodicity and the expected enrichment of ribosome-protected fragments at approximately 30–34 nucleotides. Summary quality-control reports were compiled using MultiQC.

##### Gene-set definitions

Mutant-p53 CLIP 3′UTR-bound transcripts were defined from the differential CLIP-seq peak tables (see CLIP-seq methods) as genes carrying a 3′UTR peak with greater mean signal in the mutant than in wild-type cells. Mitochondrial transcripts were defined by membership of the MitoCarta3.0 inventory.

##### Enrichment analysis

Enrichment of CLIP 3′UTR-bound and mitochondrial transcripts among altered transcripts (and, separately, among transcripts with increased occupancy) was assessed against all quantified transcripts by two-sided Fisher’s exact test. Odds ratios are reported with 95% confidence intervals calculated by the Woolf method (ln OR ± 1.96 × √(1/a +1/b + 1/c + 1/d)).

##### Fold-change distributions

The distribution of Ribo-seq log_2_ fold changes for CLIP 3′ UTR-bound transcripts was compared with that of all quantified transcripts using the two-sample Kolmogorov-Smirnov test.

##### Direction of change

Within each gene set (all transcripts, CLIP 3′UTR-bound, and mitochondrial), transcripts were classified as increased, decreased, or unchanged (adjusted *p* < 0.05 and the sign of the fold change) and reported as a percentage of transcripts tested in that set.

Refer to Table S7 for the full dataset.

### QUANTIFICATION AND STATISTICAL ANALYSIS

Statistical details for each experiment are reported in the figure legends, including exact *n* values and the statistical test applied. A summary of approaches is provided below.

#### Proximity labeling proteomics

Three independent biological replicates, each with two technical replicates, were analyzed by DIANN as described in the method details. Peptide and protein intensities were log_2_-transformed and median-normalized in Perseus (v2.0.7.0). Missing values were imputed using default Perseus settings. Statistical significance was assessed by software calculated FDR (threshold = 0.05); results are displayed as volcano plots. GO term enrichment was performed using Metascape and STRING.

#### Image analysis (PLA and IF)

For each condition, more than or equal to 3 images were acquired from different regions on the coverslip. For PLA assays assessing p53 proximity to dsRNA, ZC3H7B, and COX7C, images were taken from 1 biological replicate. For localization IF assays and mitochondrial marker assays, images were taken from 2 biological replicates. For PLA assays assessing p53 proximity to TOMM70 and AKAP1, images were taken from 3 biological replicates. Puncta counts and fluorescence intensity were quantified using CellProfiler (v4.2.8) and Fiji (v1).

#### RNA-seq

Three biological replicates per condition. One replicate from the WT condition was excluded due to poor sample quality and low reads. Differential expression was performed using edgeR (v4.0.16) with TMM normalization; low-count genes were removed using edgeRfilterByExpr with default parameters. Gene set enrichment was performed using GSEApy (v0.12) with the MSigDB Hallmark gene set collection.

#### miRNA-seq

Three biological replicates per condition. Differential expression was performed using DESeq2 (v1.34.0). miRNAs with total sum count <10 across all replicates were excluded. Statistical significance was defined as adjusted Padj <0.1 for both mature miRNA and hairpin analyses.

#### CLIP-seq

Two biological replicates per condition. Uniquely mapped CLIP tags from all samples were pooled, and peaks were identified using tag2peak.pl (CTK) with a significance cutoff of *p* < 0.05. For each sample, tag counts within significant peaks were quantified using the countOverlaps function in R. Differentially bound peaks between mutant and wild-type p53 were identified using DESeq, applying an adjusted *p*-value threshold of <0.05.

#### Ribo-seq

Three biological replicates per condition. Enrichment of CLIP-bound and mitochondrial transcripts among translationally altered transcripts was assessed by two-sided Fisher’s exact test; odds ratios are reported with 95% confidence intervals calculated by the Woolf method. The distribution of log_2_ fold changes for CLIP 3′UTR-bound transcripts versus all quantified transcripts was compared using the two-sample Kolmogorov-Smirnov test. Differential translation significance was defined as adjusted *p* < 0.05.

#### TCGA pan-cancer analysis

For each miRNA and mRNA, expression was modeled by ordinary least squares as: value ~ C(TP53 status) + C(cancer type), with cancer type as a covariate to control for tissue of origin. The reported coefficient (β) represents the mean expression shift in DBD-missense versus wild-type tumors.

#### Seahorse XF assay

OCR values were normalized to cell count per well determined by Hoechst staining and InCarta image analysis. For each condition, *n* = 24 technical replicate wells were analyzed within one representative experiment; a similar trend was observed in an independent repeat experiment. Data are presented as mean ± SEM. Statistical comparisons were performed using GraphPad Prism with oneway ANOVA followed by pairwise comparisons.

#### General

Unless otherwise stated, data are presented as mean ± SEM. Statistical significance thresholds and exact *n* values are indicated in each figure legend. No randomization or blinding was applied; no formal sample size estimation was performed as all experiments used established biochemical and cell biological assays. No data were excluded from analysis unless noted.

## Supplementary Material

1

2

3

4

5

6

7

8

Supplemental information can be found online at https://doi.org/10.1016/j.celrep.2026.117856.

## Figures and Tables

**Table:** KEY RESOURCES TABLE

REAGENT or RESOURCE	SOURCE	IDENTIFIER
Antibodies		
Anti-FLAG (ms)	Cell Signaling	8146S; RRID: AB_10950495
Anti-FLAG (rb)	Cell Signaling	14793S; RRID: AB_2572291
Anti-FLAG (For IP)	Sigma Aldrich	F1804; RRID: AB_262044
Anti-HA	Cell Signaling	3724S; RRID: AB_1549585
EGF Receptor	Cell Signaling	4267S; RRID: AB_2895042
c-JUN	Cell Signaling	9165S; RRID: AB_2130165
GAPDH	Cell Signaling	97166S; RRID: AB_2756824
ZC3H7B Poly Ab	Proteintech	25624-1-AP; RRID: AB_2880166
dsRNA(K1) Mouse mAb	Cell Signaling	28764S; RRID: AB_2756865
COX7C Ab	Invitrogen	PA5-68018; RRID: AB_2691718
TOMM70 Ab	Proteintech	14528-1-AP; RRID: AB_2303727
AKAP1 Ab	Proteintech	15618-1-AP; RRID: AB_2225484
Goat Anti-Rabbit IgG H&L (Alexa Fluor^®^ 488)	Abcam	ab150077; RRID: AB_2630356
Goat Anti-Rabbit IgG H&L (Alexa Fluor^®^ 555)	Abcam	ab150078; RRID: AB_2722519
Goat Anti-Rabbit IgG H&L (Alexa Fluor^®^ 647)	Abcam	ab150079; RRID: AB_2722623
Goat Anti-Mouse IgG H&L (Alexa Fluor^®^ 488)	Abcam	ab150113; RRID: AB_2576208
Goat Anti-Mouse IgG H&L (Alexa Fluor^®^ 555)	Abcam	ab150114; RRID: AB_2687594
Goat Anti-Mouse IgG H&L (Alexa Fluor^®^ 647)	Abcam	ab150115; RRID: AB_2687948
Goat Anti Rabbit 680 (Li-Cor)	LICORbio	926-68071; RRID: AB_10956166
Goat Anti Rabbit 800 (Li-Cor)	LICORbio	926-32211; RRID: AB_621843
Goat Anti Mouse 680 (Li-Cor)	LICORbio	926-68070; RRID: AB_10956588
Streptavidin IRDye 800CW (Li-Cor)	LICORbio	925-32230
Goat Anti Rabbit IgG (H + L) Alexa 488	Thermo Fisher	A32731; RRID: AB_2633280
Goat Anti Mouse IgG (H + L) Alexa 488	Thermo Fisher	A32723; RRID: AB_2633275
Bacterial and virus strains		
Lentivirus expressing p53 variants (WT, G245S, and R273H)	VectorBuilder	N/A
NEB^®^ 5-alpha Competent E. coli	New England Biolabs	C2987
Chemicals, peptides, and recombinant proteins		
CfaC-Ir (μMap photocatalyst)	MacMillan Laboratory, Princeton University	N/A
diazirine-biotin	In house synthesis	N/A
Cycloheximide	Millipore Sigma	Cat#C1988
RIPA Lysis Buffer (10X)	Millipore Sigma	Cat#20-188
Halt Protease Inhibitor Cocktail (100X)	Thermo Fisher Scientific	Cat#78438
Pierce BCA Protein Assay Kit	Thermo Fisher Scientific	Cat#23227
Lipofectamine 2000	Thermo Fisher Scientific	Cat#11668019
DNA/RNA Shield	Zymo Research	Cat#1100-50
RNeasy Plus Mini Kit	Qiagen	Cat#74134
SUPERase-In RNase Inhibitor	Thermo Fisher Scientific	Cat#AM2694
TURBO DNase	Thermo Fisher Scientific	Cat#AM2239
MitoTracker Red	Thermo Fisher Scientific	Cat# M22426
MitoTracker Green	Thermo Fisher Scientific	Cat# M7514
MitoTracker Orange	Thermo Fisher Scientific	Cat#M7511
Poly-L-lysine	Sigma Aldrich	Cat#P4707
Phusion Polymerase	New England Biolabs	Cat#M0536S
Sera-Mag SpeedBeads E7	Cytiva Life Sciences	Cat#45152105050250
Sera-Mag SpeedBeads E3	Cytiva Life Sciences	Cat#65152105050250
TRIzol Reagent	Thermo Fisher Scientific	Cat#15596026
DPBS	Gibco	Cat#14190250
Fetal Bovine Serum	Gibco	Cat#10437-028
Penicillin/Streptomycin	Gibco	Cat#15140-122
Trypsin-EDTA	Gibco	Cat#25300054
Trypsin Protease MS grade	Thermo Fisher Scientific	Cat#90057
Laemmli Sample Buffer (4x)	Bio-Rad	Cat#1610747
Protein A Magnetic Beads	Thermo Fisher Scientific	Cat#88845
Dynabeads Protein G	Invitrogen	Cat #10003D
4% Paraformaldehyde in PBS	Thermo Fisher Scientific	Cat#AAJ61899AP
DAPI mounting media	Fisher Scientific	Cat#NC9524612
Critical commercial assays		
Subcellular Protein Fractionation Kit for Cultured Cells	Thermo Fisher Scientific	Cat#78840
Duolink PLA kit (anti-Rabbit PLUS Probe)	Sigma Aldrich	DUO92002
Duolink PLA kit (anti-Mouse MINUS Probe)	Sigma Aldrich	DUO92004
Duolink PLA kit (reagent red)	Sigma Aldrich	DUO92007
Seahorse XFe96/XF Pro FluxPak	Agilent Technologies	Cat#103792-100
Qubit RNA BR Assay Kit	Thermo Fisher Scientific	Cat#Q10210
Qubit ssDNA Assay Kit	Thermo Fisher Scientific	Cat#Q10212
NEXTFLEX Small RNA Kit v4	Revvity	NOVA-5132-31
Deposited data		
Mass spectrometry raw data	MassIVE	MassIVE: MSV000099926
CLIP-seq data	NCBI GEO	GSE310613
RNA-seq data	NCBI GEO	GSE310614
miRNA-seq data	NCBI GEO	GSE310615
Ribo-seq data	NCBI GEO	GSE335668
Experimental models: Cell lines		
HEK293T	ATCC	Cat#CRL-3216
HCT116	Abcam	ab289184
HCT116 p53 KO	Abcam	ab289184
Oligonucleotides		
COX7C forward: CTTCCAGCAGCGGTATGTT	Integrated DNA Technologies	N/A
COX7C reverse: AGCTAGTAACGACCACTTGTTT	Integrated DNA Technologies	N/A
GAPDH forward: AATCCCATCACCATCTTCCAG	Integrated DNA Technologies	N/A
GAPDH reverse: CCTTCTCCATGGTGGTGAAGAC	Integrated DNA Technologies	N/A
Recombinant DNA		
pcDNA3.1(+)-H3.1-CfaN backbone	Gift from D.W.C. MacMillan lab	N/A
pcDNA3.1(+)-H3.1-CfaN p53 variants	Cloned in house	N/A
Software and algorithms		
DIANN 1.8.1	Demichev et al., 2020	https://github.com/vdemichev/DiaNN
Perseus v2.0.7.0	Tyanova et al., 2016	https://maxquant.net/perseus/
Metascape	Zhou et al., 2019	https://metascape.org
STRING v12	Szklarczyk et al., 2023	https://string-db.org
CLIP Tool Kit (CTK)	Shah et al., 2017	https://github.com/chaolinzhanglab/ctk
BWA	Li & Durbin, 2009	https://github.com/lh3/bwa
STAR v2.7.11	Dobin et al., 2013	https://github.com/alexdobin/STAR
featureCounts/Subread v2.1.1	Liao et al., 2014	https://subread.sourceforge.net
edgeR v4.0.16	Robinson et al., 2010	https://bioconductor.org/packages/edgeR
DESeq2 v1.34.0	Love et al., 2014	https://bioconductor.org/packages/DESeq2
nf-core/smrnaseq	nf-core	https://nf-co.re/smrnaseq
Nextflow v25.04.4	Di Tommaso et al., 2017	https://www.nextflow.io
Trim Galore/Cutadapt	Martin, 2011	https://github.com/FelixKrueger/TrimGalore
Ribo-TISH	Zhang et al., 2017	https://github.com/zhpn1024/ribotish
Ribotricer	Choudhary et al., 2020	https://github.com/smithlabcode/ribotricer
FastQC v0.12.1	Andrews, 2010	https://www.bioinformatics.babraham.ac.uk/projects/fastqc
MultiQC v1.32	Ewels et al., 2016	https://multiqc.info
Wave v2.6.4.39	Agilent Technologies	https://www.agilent.com/en/product/cell-analysis/real-time-cell-metabolic-analysis/xf-software/seahorse-wave-desktop-software-740897
MetaXpress Acquire Software 2025.1	Molecular Devices	https://www.moleculardevices.com/products/cellular-imaging-systems/acquisition-and-analysis-software/metaxpress
InCarta v2.8	Molecular Devices	https://www.moleculardevices.com/products/cellular-imaging-systems/acquisition-and-analysis-software/incarta-software
Fiji v1	Schneider et al., 2012	https://imagej.net
CellProfiler 4.2.8	Stirling et al., 2021	https://cellprofiler.org
SnapGene 7.2	GSL Biotech	https://www.snapgene.com
R/RStudio	R Core Team	https://www.r-project.org
Python (pandas, numpy, scipy, statsmodels)	Python Software Foundation	https://www.python.org

## Highlights

- **μ**Map photoproximity labeling maps *in situ* interactomes of five p53 hotspot mutants
- Mutant p53 gains RNA-scaffolded protein interactions
- Mutant p53 binds 3′UTRs and remodels miRNA processing
- Ribosome profiling links mutant p53 RNA binding to altered translation
