# Supplementary material for: Mutant p53 gains RNA-binding activity to reprogram translation and mitochondrial function
Source: Cell Rep. Author manuscript; Available in PMC 2026 Sep 10. (PMC13559719; doi:10.1016/j.celrep.2026.117856)
Supplement: Supplementary file 1 [file NIHMS2206935-supplement-1.pdf]

Cell Reports, Volume *45*

Supplemental information

Mutant p53 gains RNA-binding activity to reprogram translation and mitochondrial function

Wuyue Zhou, Alice Long, Cameron J. Douglas, Dalila Gallegos, James M. Burke, David W. C. MacMillan, Ezgi Hacisuleyman, and Ciaran P. Seath

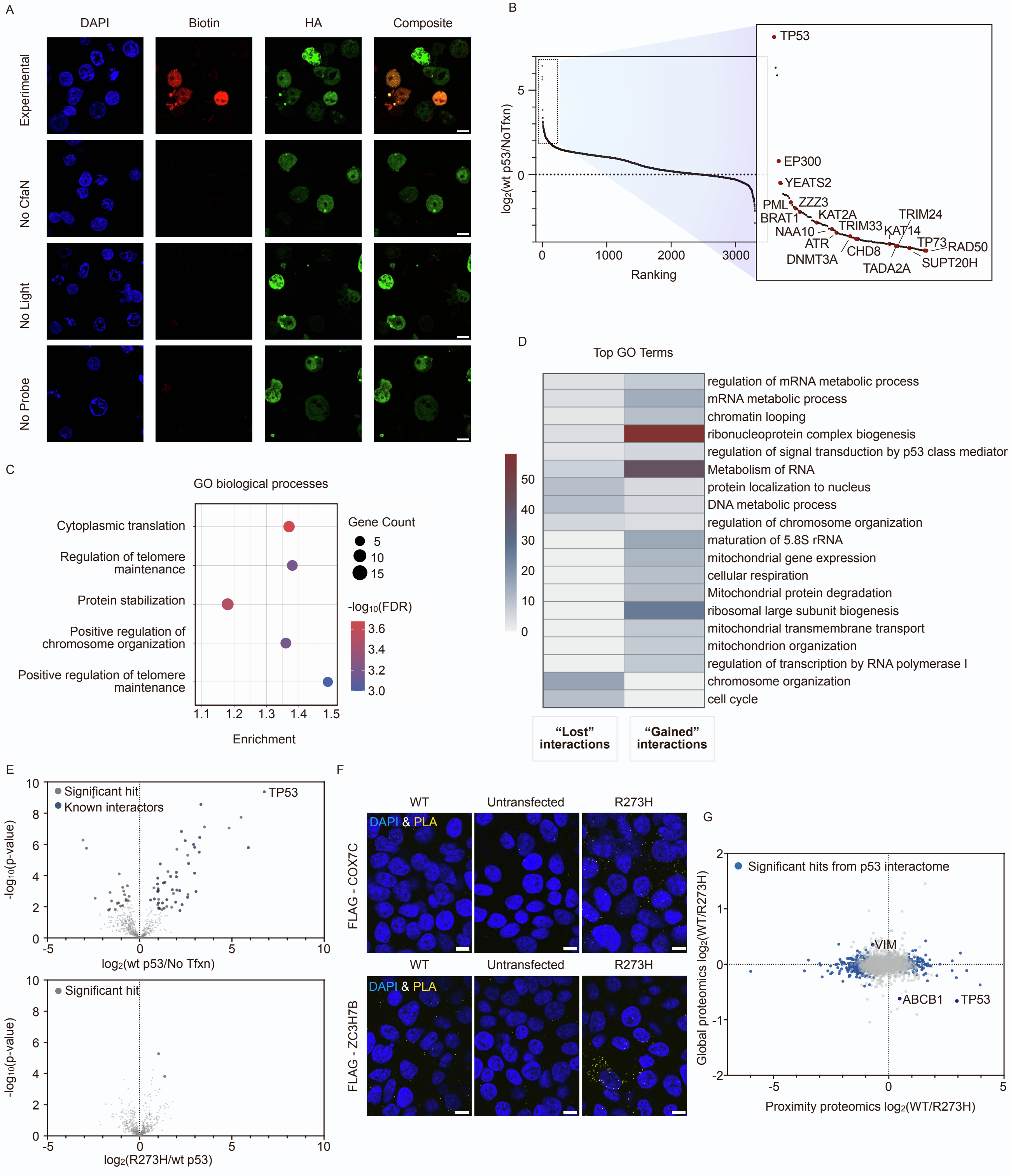
A
DAPI
Biotin
HA
Composite
Experimental
No CfaN
No Light
No Probe
B
log2(wt p53/NoTfxn)
Ranking
TP53
EP300
YEATS2
PML
ZZZ3
BRAT1
KAT2A
TRIM24
NAA10
TRIM33
KAT14
ATR
TP73
RAD50
DNMT3A
CHD8
TADA2A
SUPT20H
C
GO biological processes
Cytoplasmic translation
Regulation of telomere maintenance
Protein stabilization
Positive regulation of chromosome organization
Positive regulation of telomere maintenance
Enrichment
Gene Count
-log10(FDR)
D
Top GO Terms
regulation of mRNA metabolic process
mRNA metabolic process
chromatin looping
ribonucleoprotein complex biogenesis
regulation of signal transduction by p53 class mediator
Metabolism of RNA
protein localization to nucleus
DNA metabolic process
regulation of chromosome organization
maturation of 5.8S rRNA
mitochondrial gene expression
cellular respiration
Mitochondrial protein degradation
ribosomal large subunit biogenesis
mitochondrial transmembrane transport
mitochondrion organization
regulation of transcription by RNA polymerase I
chromosome organization
cell cycle
"Lost" interactions
"Gained" interactions
E
Significant hit
Known interactors
TP53
-log10(p-value)
log2(wt p53/No Tfxn)
Significant hit
log2(R273H/wt p53)
F
WT
Untransfected
R273H
DAPI & PLA
FLAG - COX7C
FLAG - ZC3H7B
G
Significant hits from p53 interactome
VIM
ABCB1
TP53
Global proteomics log2(WT/R273H)
Proximity proteomics log2(WT/R273H)

Figure S1: μMap labeling is validated and reveals distinct interactome changes upon R273H mutation. (A) Immunofluorescence detection of biotinylation signal in permeabilized cells, validating μ-Map labeling specificity. Scale bar = 10 μm. (B) Waterfall plot with known p53 interactors annotated in the wt p53 μ-Map interactome. Wild-type p53 was among the top hits. (C) GO biological processes of the wt p53 interactome. Terms are ranked by a weighted harmonic mean between the enrichment score and $-\log_{10}$FDR. (D) GO analysis of proteins recruited to mutant R273H p53 ("gained" interactions) and proteins repulsed from mutant ("lost" interactions). (E) IP-MS of p53. Top: A volcano plot derived from a two-sided t-test showing wt p53 interactors from IP-MS. Wild-type p53 was among the top hits, and known interactors were labeled. FDR<0.05. Bottom: A volcano plot derived from a two-sided t-test showing "gained" interactions and "lost" interactions of R273H p53 comparing to wt p53 from IP-MS. Few differential interactions were observed. (F) Representative images from PLA assay shown in Fig. 2G, H. Scale bar = 10 μm. (G) Scatter plots of μ-Map obtained p53 interactome shift (x-axis) and whole cell proteome (y-axis) changes in response to p53 mutation (R273H). Genes of interest are labeled and highlighted in blue.

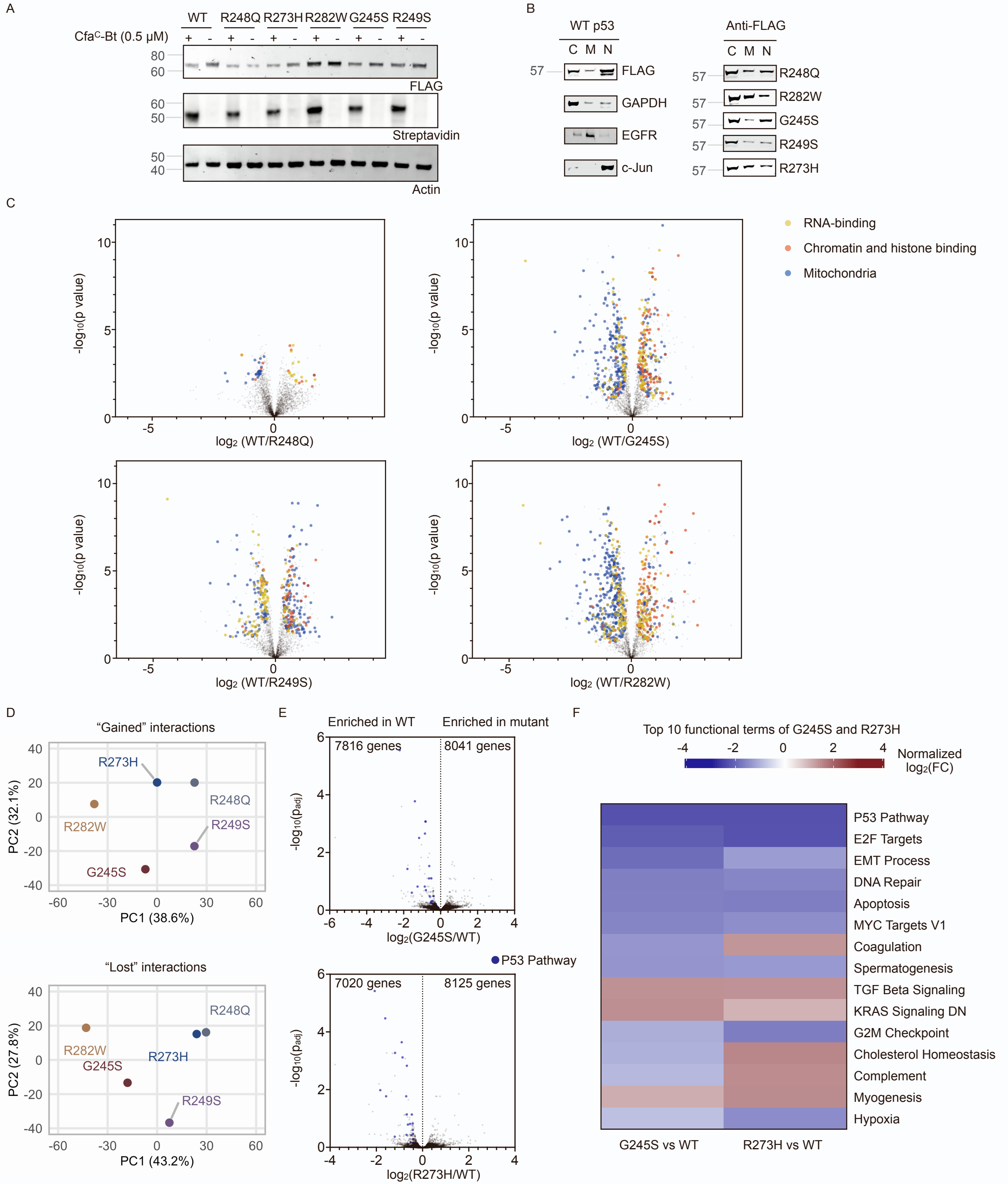

A
WT R248Q R273H R282W G245S R249S
Cfa^C-Bt (0.5 μM)
FLAG
Streptavidin
Actin
B
WT p53
Anti-FLAG
C M N
FLAG
GAPDH
EGFR
c-Jun
R248Q
R282W
G245S
R249S
R273H
C
RNA-binding
Chromatin and histone binding
Mitochondria
-log10(p value)
log2 (WT/R248Q)
log2 (WT/G245S)
log2 (WT/R249S)
log2 (WT/R282W)
D
"Gained" interactions
"Lost" interactions
PC1 (38.6%)
PC2 (32.1%)
PC1 (43.2%)
PC2 (27.8%)
E
Enriched in WT
Enriched in mutant
7816 genes
8041 genes
-log10(padj)
log2(G245S/WT)
P53 Pathway
7020 genes
8125 genes
log2(R273H/WT)
F
Top 10 functional terms of G245S and R273H
Normalized log2(FC)
P53 Pathway
E2F Targets
EMT Process
DNA Repair
Apoptosis
MYC Targets V1
Coagulation
Spermatogenesis
TGF Beta Signaling
KRAS Signaling DN
G2M Checkpoint
Cholesterol Homeostasis
Complement
Myogenesis
Hypoxia
G245S vs WT
R273H vs WT

Figure S2: p53 hotspot mutants show cytoplasmic mislocalization and distinct interactome and transcriptome signatures. (A) The spliced product is only observed while treated with Cfa^{C}-Bt for all p53 mutants used in this study. (B) Mutant and wt p53 distribution revealed by cellular fractionation. C= cytosol, M= cell membrane, and N= nucleus. Corresponding markers were also stained. (C) Interactome shift caused by p53 mutation. All volcano plots were derived from a two-sided t-test comparing wt p53 interactors to mutant p53 interactors. FDR<0.05. RNA-binding, chromatin and histone binding, and mitochondrial proteins are annotated. (D) PCA analysis of gene annotations of p53 mutants. "Lost" and "gained" interaction proteins for each p53 hotspot mutant (R273H, R248Q, R282W, G245S, R249S) compared to wild-type p53 were submitted to Metascape for gene ontology (GO) enrichment analysis. The resulting –log10(p-value) scores across all GO terms were used as input features for principal component analysis (PCA). (E) Transcriptome change caused by mutant p53 expression. Volcano plots were derived from a two-sided t-test comparing transcripts in mutant p53 expressing cells to transcripts in wt p53 expressing cells. Number of enriched genes is annotated. Genes relevant to the p53 pathway are down-regulated in both mutant forms of p53, and are annotated on the graph. (F) Combined top functional terms of differentially regulated genes in both mutants. Entries were ranked by adjusted p-values in ascending order.

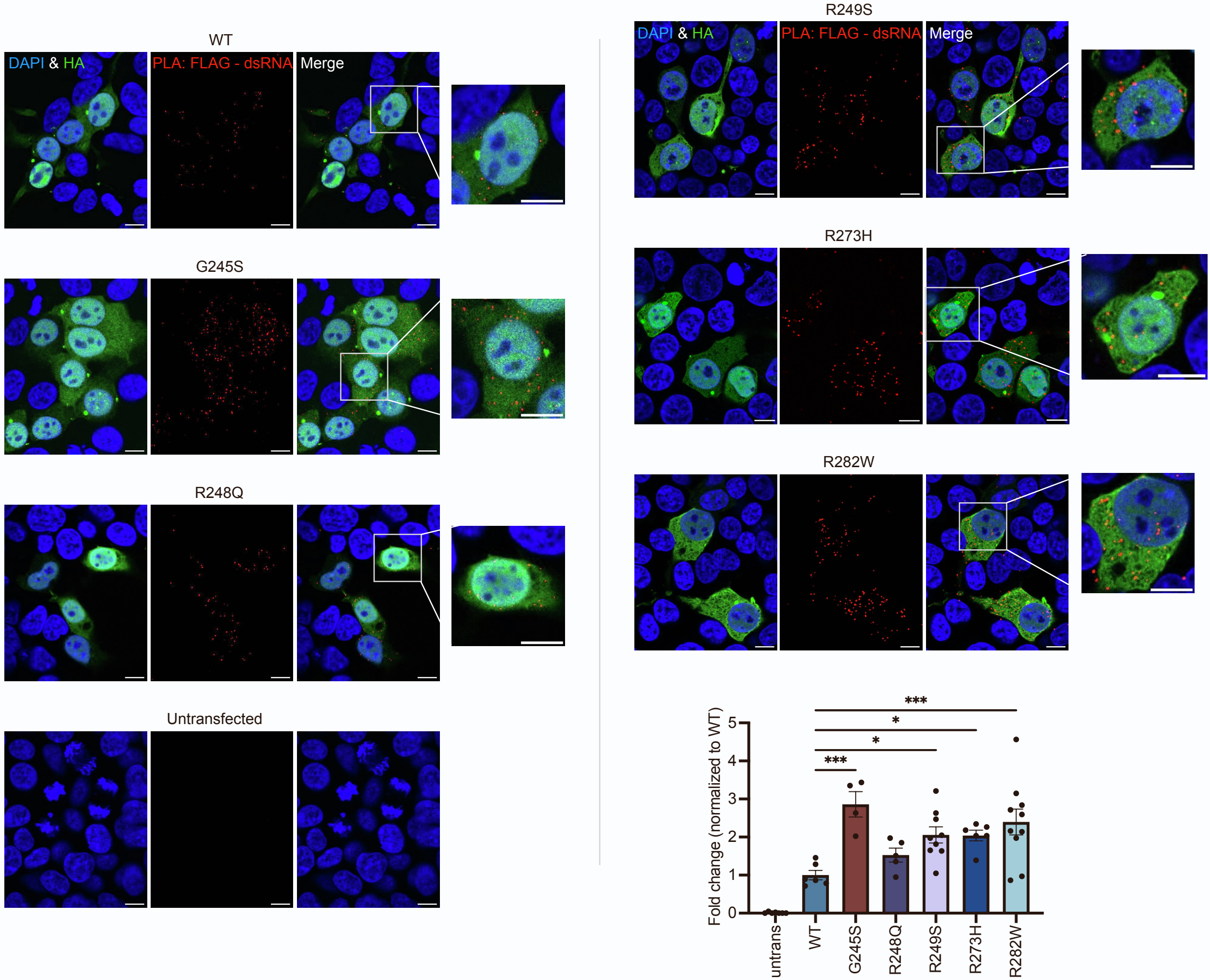

Figure S3: Representative images from PLA assay testing expressed p53 construct (FLAG) interaction with dsRNA in Fig. 4C. Bar graph depicting puncta quantification (n ≥ 4, one-way ANOVA test followed by post hoc Dunnett's multiple comparisons comparing to untransfected control. *P<0.05, ***P<0.001). PLA puncta per field of view were quantified for analysis (mean ± SEM). HA immunostaining identified transfected cells. Scale bar = 10 μm.

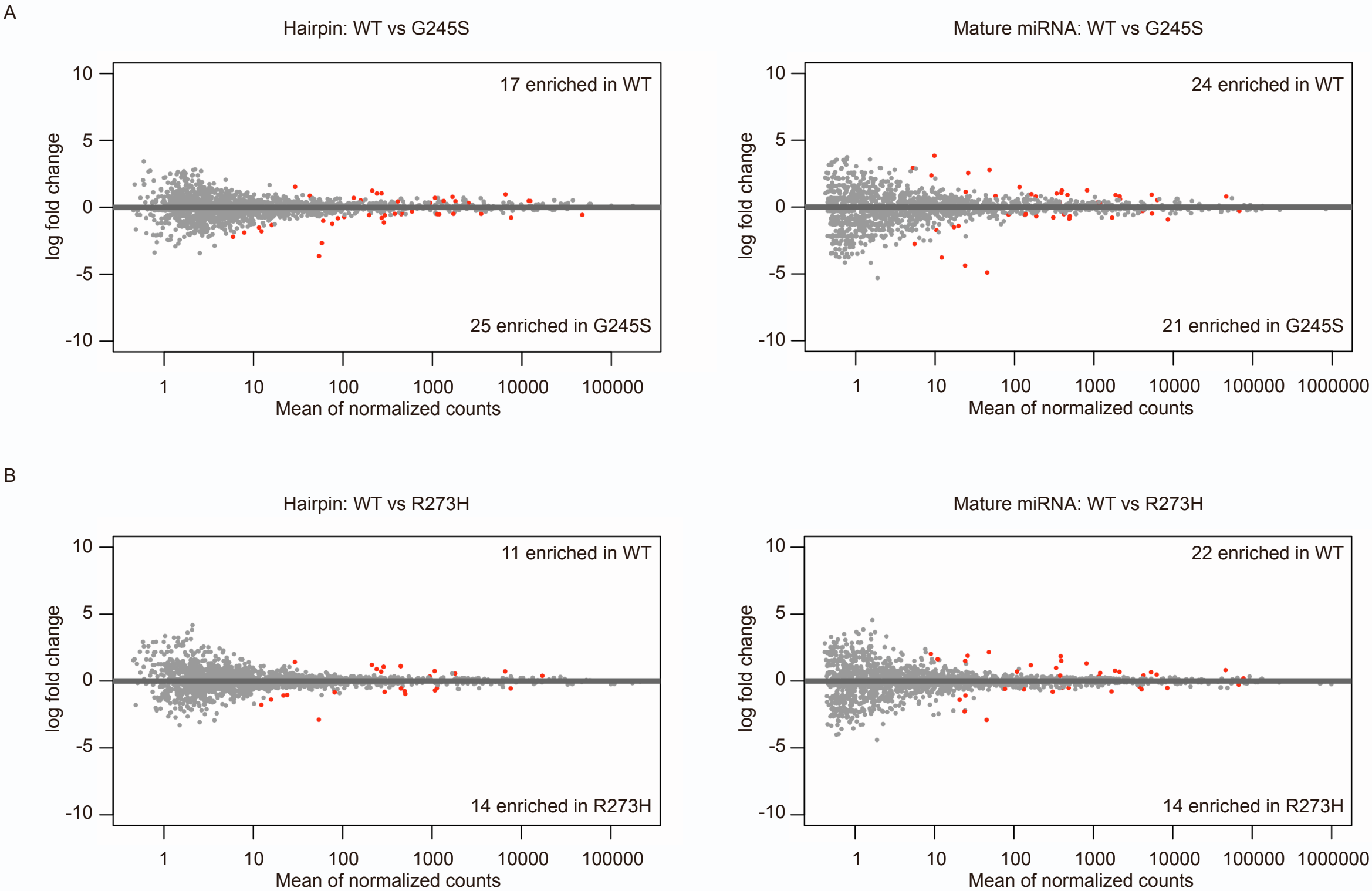

Figure S4: MA plots reveal mutant p53-driven changes in the miRNA landscape. (A) MA plot derived from DESeq2. Differentially regulated mature miRNA or hairpins in G245S mutant were highlighted. Adjusted p-value<0.1. (B) MA plot derived from DESeq2. Differentially regulated mature miRNA or hairpins in R273H mutant were highlighted. Adjusted p-value<0.1.

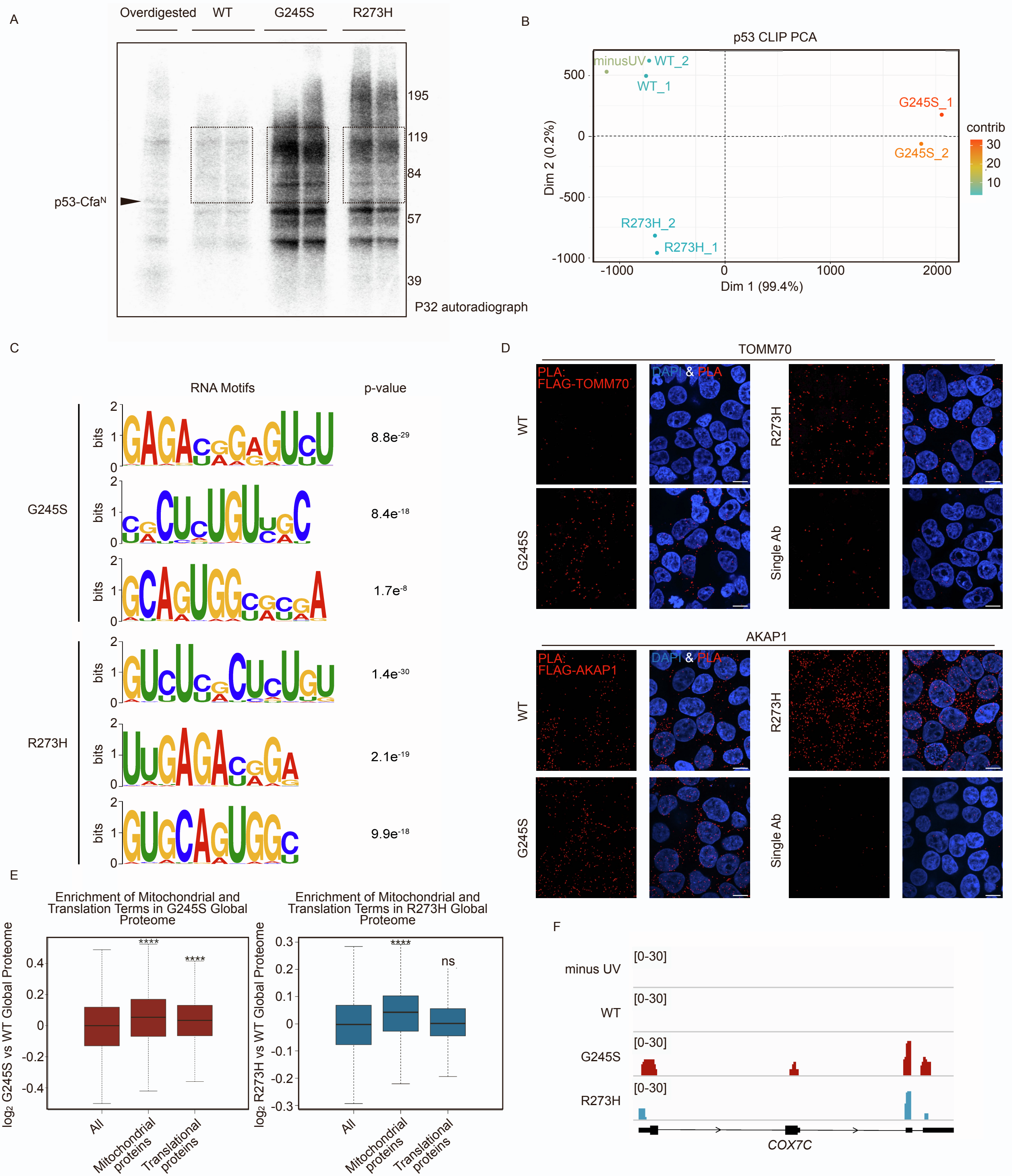
A
Overdigested
WT
G245S
R273H
195
119
84
57
39
p53-Cfa^N
P32 autoradiograph
B
p53 CLIP PCA
minusUV
WT_2
WT_1
G245S_1
G245S_2
R273H_2
R273H_1
contrib
30
20
10
Dim 2 (0.2%)
Dim 1 (99.4%)
500
0
-500
-1000
-1000
0
1000
2000
C
RNA Motifs
p-value
G245S
R273H
bits
8.8e-29
8.4e-18
1.7e-8
1.4e-30
2.1e-19
9.9e-18
D
TOMM70
PLA:
FLAG-TOMM70
DAPI & PLA
WT
G245S
R273H
Single Ab
AKAP1
PLA:
FLAG-AKAP1
DAPI & PLA
WT
G245S
R273H
Single Ab
E
Enrichment of Mitochondrial and Translation Terms in G245S Global Proteome
log2 G245S vs WT Global Proteome
****
****
All
Mitochondrial proteins
Translational proteins
Enrichment of Mitochondrial and Translation Terms in R273H Global Proteome
log2 R273H vs WT Global Proteome
****
ns
All
Mitochondrial proteins
Translational proteins
F
minus UV
WT
G245S
R273H
[0-30]
[0-30]
[0-30]
[0-30]
COX7C

Figure S5: CLIP-seq quality metrics and proteomic data support mutant p53 binding at the mitochondrial surface. (A) Representative autoradiograph image of CLIP-seq samples showing RNAs bound by wild type and mutant p53 after pull-down. Regions corresponding to p53 binding (labeled by dashed lines) were cut and subjected to digestion and purification to acquire RNA. (B) PCA plot showing CLIP-seq sample distribution. (C) Other consensus RNA-binding motifs of mutant p53 identified by STREME analysis. (D) Representative images from PLA assay testing expressed p53 construct (FLAG) interaction with TOMM70 or with AKAP1. Scale bar = 10 µm. (E) Among mutant p53 proteome, proteins associated with mitochondria (G245S and R273H) and translation (G245S) were highly enriched. Mitochondrial proteins were obtained from MitoCarta 3.0, and translation-related proteins were retrieved from MSigDB (GOBP: Translation; GO:0006412). Statistical significance was calculated using the Wilcoxon signed-rank test. (F) Visualization of CLIP-seq read coverage on transcript *COX7C*.

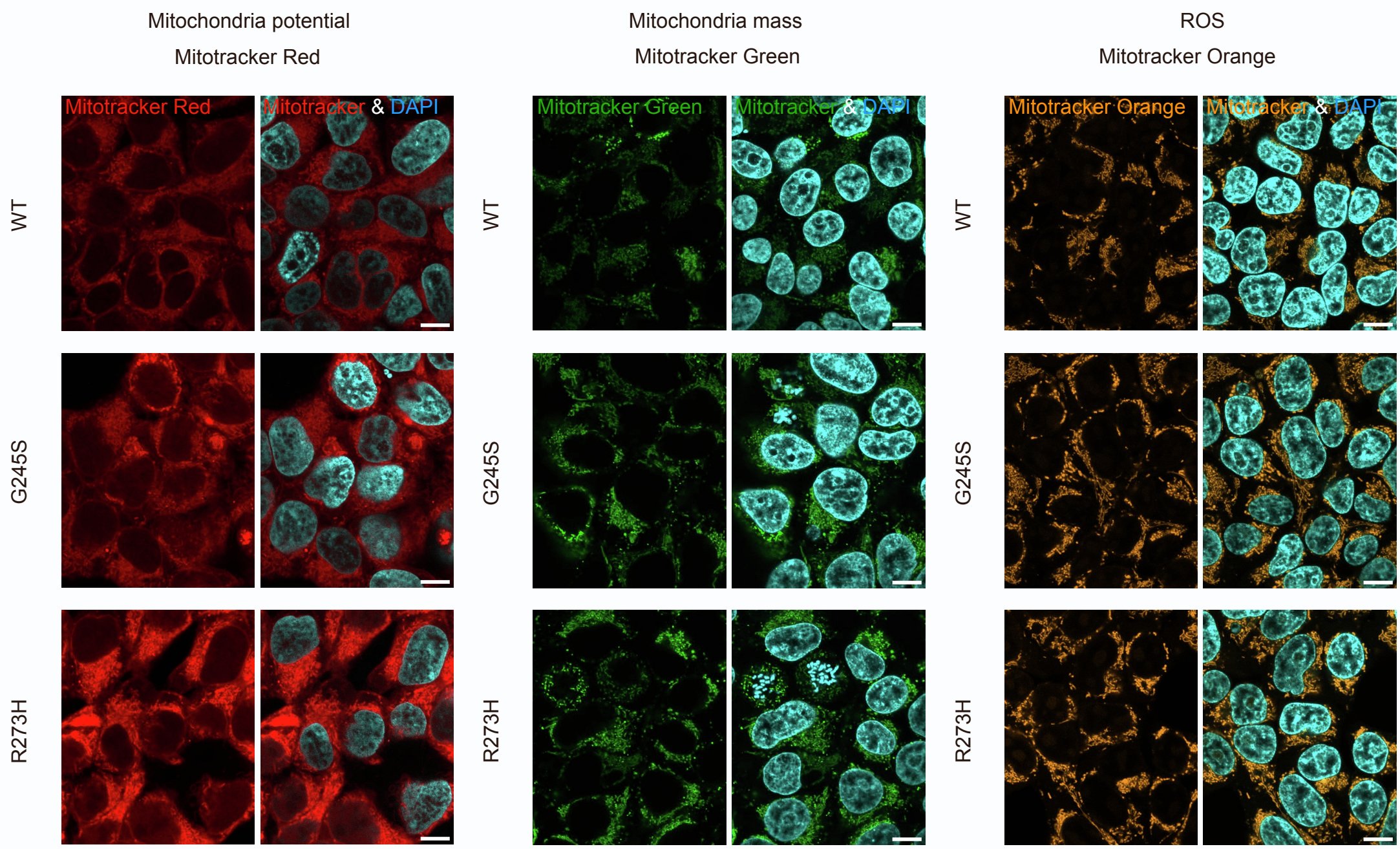

Figure S6: Representative images of Mitotracker assay. Scale bar = 10 μm.

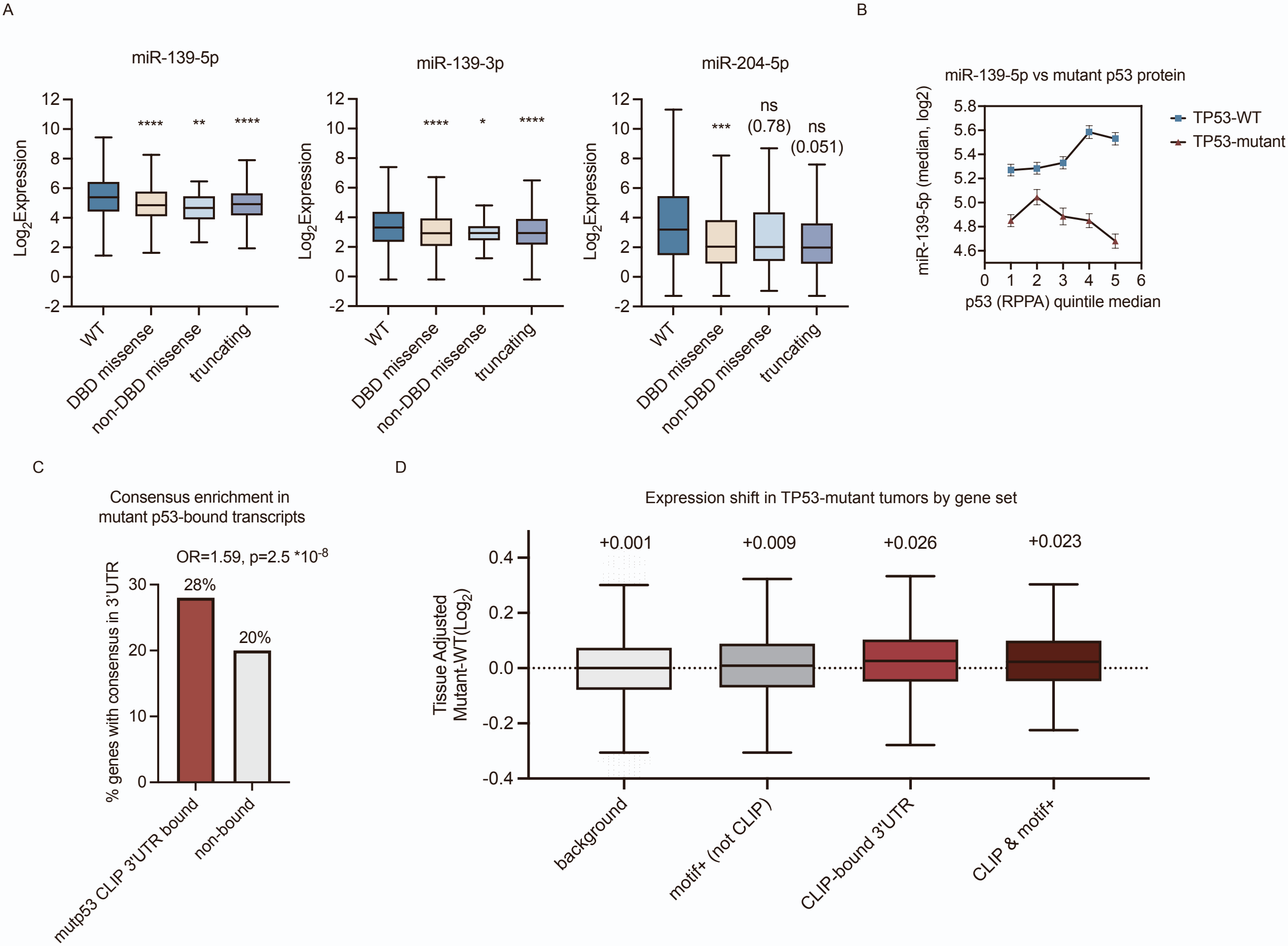
A
miR-139-5p
miR-139-3p
miR-204-5p
Log2Expression
WT
DBD missense
non-DBD missense
truncating
****
**
*
***
ns (0.78)
ns (0.051)
B
miR-139-5p vs mutant p53 protein
miR-139-5p (median, log2)
p53 (RPPA) quintile median
TP53-WT
TP53-mutant
C
Consensus enrichment in mutant p53-bound transcripts
OR=1.59, p=2.5 *10-8
% genes with consensus in 3'UTR
28%
20%
mutp53 CLIP 3'UTR bound
non-bound
D
Expression shift in TP53-mutant tumors by gene set
Tissue Adjusted Mutant-WT(Log2)
+0.001
+0.009
+0.026
+0.023
background
motif+ (not CLIP)
CLIP-bound 3'UTR
CLIP & motif+

Figure S7: Mutant p53 RNA targets and tumor-suppressor miRNAs track *TP53* status across human tumors. Analyses span 10,323 TCGA Pan-Cancer Atlas tumors (33 cancer types); all comparisons are adjusted for cancer type (tissue). (A) Mature miRNA expression ($\log_2$) across TP53 strata — wild-type, DNA-binding-domain (DBD) missense, non-DBD missense, and truncating/null — for the tumor-suppressor miRNAs validated in our cells. miR-139-5p, miR-139-3p, and miR-204-5p are each significantly lower in mutant than wild-type tumors ($\beta = -0.23$, $\beta = -0.196$, and -0.21; $q \approx 1\times10^{-12}$, 3.5×10^{-9}, and 2×10^{-4}, respectively; tissue-adjusted linear model). The reduction spans the DBD-missense class rather than individual hotspots and is also present in truncating/null tumors, indicating a loss-of-function component. Box, interquartile range and median; whiskers, Tukey. Asterisks denote significance of each mutant stratum versus wild-type from the tissue-adjusted linear model (BH-corrected): *$q < 0.05$, **$q < 0.01$, ***$q < 0.001$, ****$q < 0.0001$; ns, not significant. (B) Median miR-139-5p expression across quintiles of p53 protein abundance (reverse-phase protein array, RPPA), in TP53-wild-type versus TP53-mutant tumors. miR-139-5p rises with p53 protein in wild-type tumors but falls with mutant-p53 protein, a mutant-specific relationship (mutant × p53 interaction $\beta = -0.22$, $P < 1\times10^{-4}$; tissue- and tumor-purity-adjusted). Points, quintile medians; error bars, SEM. (C) Percentage of genes containing the GGCUGG-centered consensus in their 3′UTR, among mutant-p53 CLIP 3′UTR-bound transcripts versus non-bound genes (odds ratio = 1.59, $P = 2.5\times10^{-8}$, Fisher's exact test). (D) Tissue-adjusted expression difference (mutant − WT, $\log_2$) for genes grouped by background, presence of the GGCUGG-centered consensus in their 3′UTR ("motif+, not CLIP"), mutant-p53 CLIP 3′UTR binding, or both. Median shift per group is indicated above each box. Transcripts bound by mutant p53 are coordinately elevated in TP53-mutant tumors, more so than motif-only or background genes. Box, interquartile range and median; whiskers, Tukey.

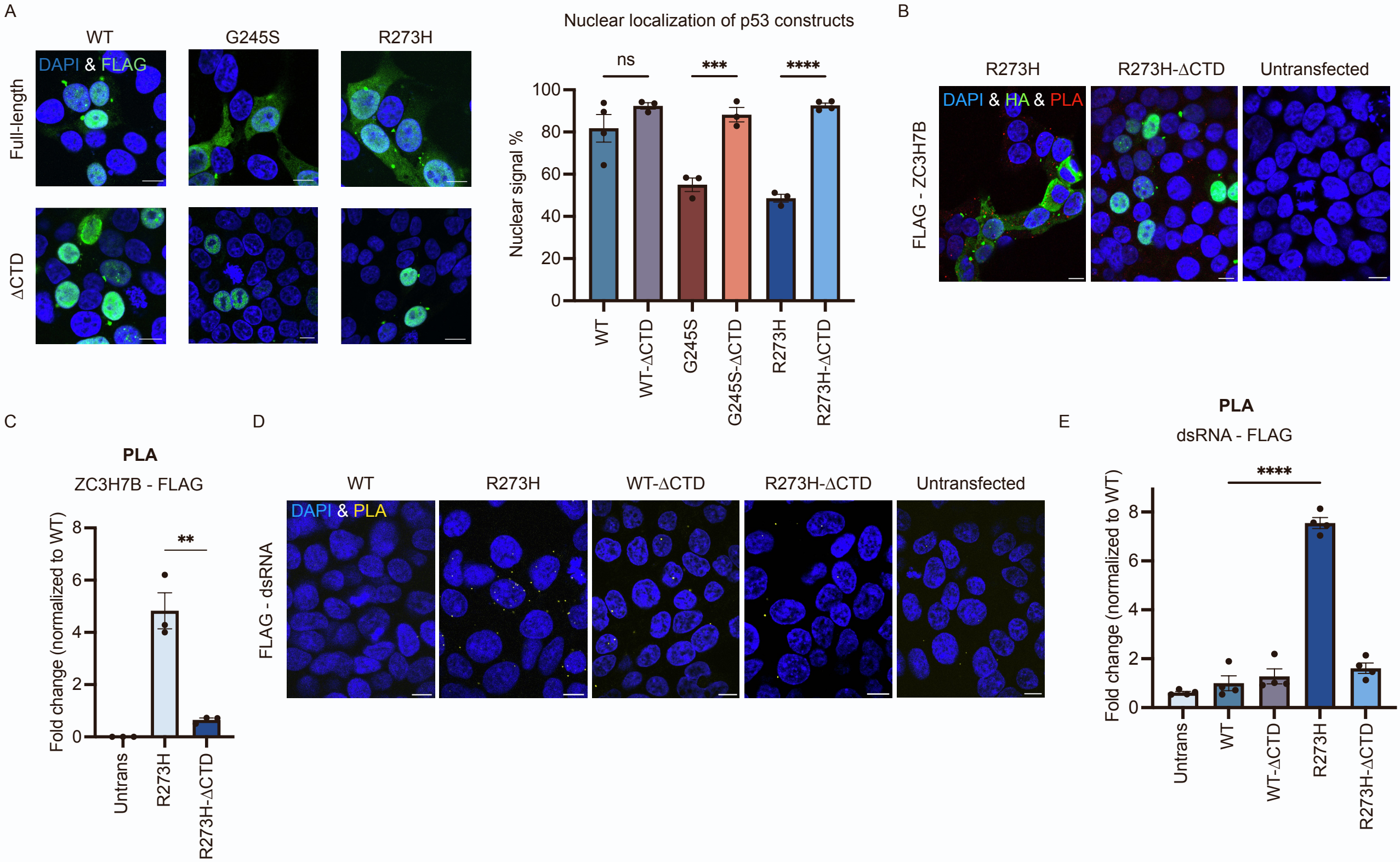

Figure S8: CTD truncation shifts p53 toward nuclear localization and reduces its RNA/protein proximity interactions. (A) Subcellular localization of full-length p53 and CTD-truncated p53 in HEK293T cells. Left: representative images, scale bar = 10 µm. Right: Deletion of the CTD domain leads to nuclear localization in both mutant and wild type p53 (n≥3, mean ± SEM, one-way ANOVA test followed by post hoc Dunnett's multiple comparisons comparing to corresponding full length control. ***P<0.001, **** P<0.0001, ns, not significant). (B) and (C): representative images (B) and quantification (C) of PLA confirmation of interaction between R273H p53 and ZC3H7B after CTD truncation (n = 3, two-sample t-test. ** P<0.01). Scale bar = 10 µm. PLA puncta per field of view were quantified for analysis (mean ± SEM). (D) and (E): representative images (D) and quantification (E) of PLA confirmation of the interaction between p53 and dsRNA after CTD truncation (n = 4, one-way ANOVA test followed by post hoc Dunnett's multiple comparisons comparing to WT control. **** P<0.0001). Scale bar = 10 µm. PLA puncta per field of view were quantified for analysis (mean ± SEM).

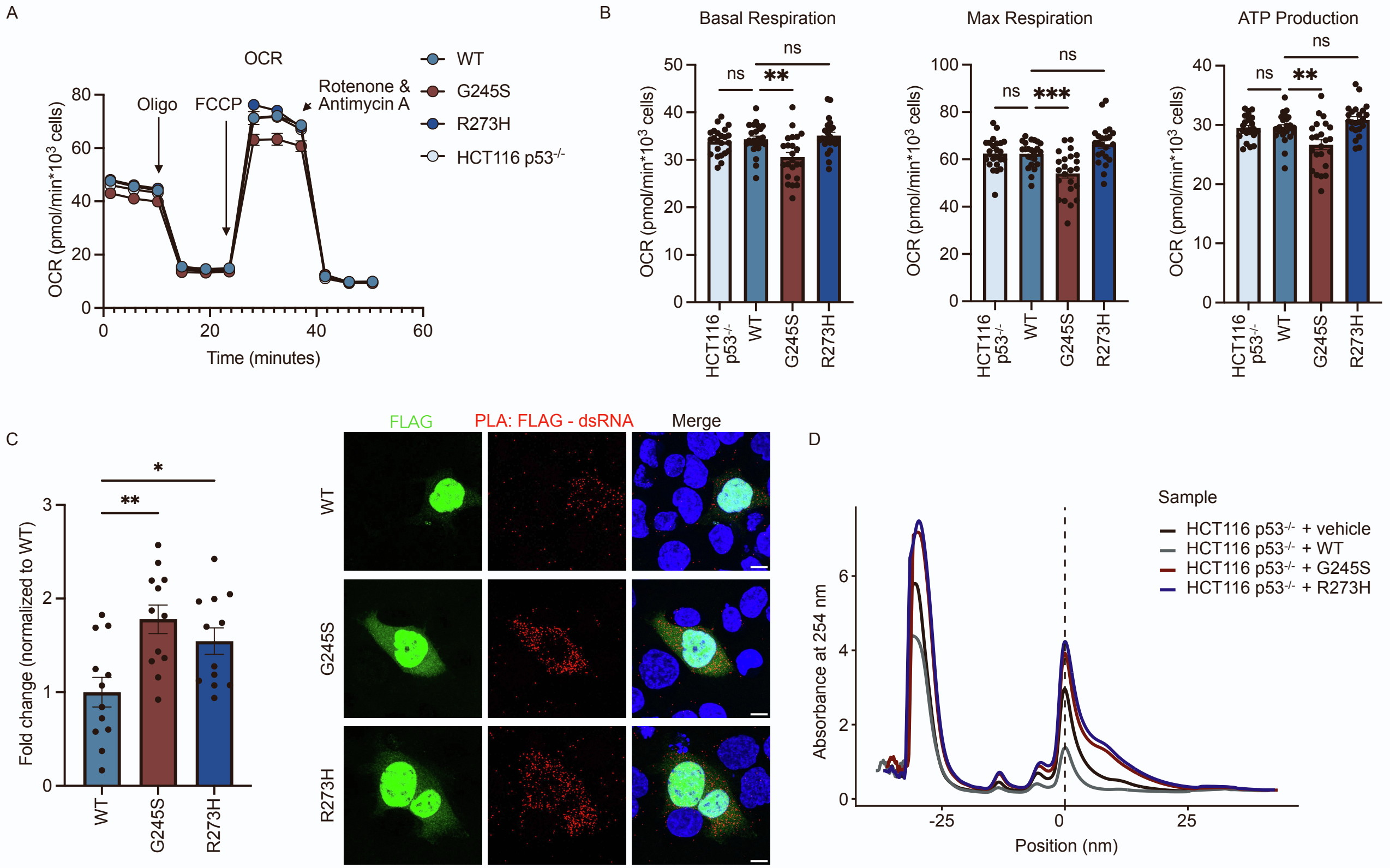

Figure S9: Mutant p53 expression recapitulates altered mitochondrial function and RNA interactions in a second cell line. (A) Mitochondrial respiration was measured by Seahorse XF assay in HCT116 p53$^{-/}$ cells transiently expressing wild-type, G245S, or R273H p53, with HCT116 p53$^{-/}$ cells transfected with vehicle included as the control. OCR (oxygen consumption rate) was recorded following sequential injection of oligomycin, FCCP, and rotenone/antimycin A. Data are normalized to cell number by Hoechst staining. (B) Quantification of basal respiration, maximal respiration, and ATP-linked respiration derived from (A). Bars show mean ± SEM. One-way ANOVA with Dunnett's multiple comparisons test relative to WT. ns, not significant; **, $p < 0.01$; ***, $p < 0.001$. (C) Proximity ligation assay (PLA) quantifying interaction between expressed p53 (FLAG) and double-stranded RNA (dsRNA; K1 antibody) in HCT116 p53$^{-/}$ cells expressing p53 variants. Left: fold change in PLA puncta per cell, normalized to WT (n ≥ 12, One-way ANOVA with Dunnett's multiple comparisons relative to WT. *, $p < 0.05$; **, $p < 0.01$). PLA puncta per field of view were quantified for analysis (mean ± SEM). Right: representative confocal images showing FLAG immunofluorescence (green), PLA signal (FLAG × dsRNA, red), and DAPI merge for WT, G245S, and R273H. Scale bar = 10 µm. (D) Monosome profiles of HCT116 p53$^{-/}$ cells transiently transfected with vehicle, wild-type, G245S, or R273H p53. Lysates were subjected to MNase digestion followed by sucrose density centrifugation; absorbance at 254 nm is plotted across gradient fractions. The dashed line marks the 80S monosome peak.
